# Contriving Multi-Epitope Subunit of Vaccine for COVID-19: Immunoinformatics Approaches

**DOI:** 10.3389/fimmu.2020.01784

**Published:** 2020-07-28

**Authors:** Rong Dong, Zhugang Chu, Fuxun Yu, Yan Zha

**Affiliations:** ^1^Department of Biomedicine, Guizhou University School of Medicine, Guiyang, China; ^2^Department of Nephrology, Guizhou Provincial People's Hospital, Guiyang, China; ^3^NHC Key Laboratory of Pulmonary Immunological Diseases (Guizhou Provincial People's Hospital), Guiyang, China; ^4^Department of Urinary Surgery, Guizhou Provincial People's Hospital, Guiyang, China

**Keywords:** immunoinformatics, epitope prediction, COVID-19, SARS-CoV-2, vaccine

## Abstract

COVID-19 has recently become the most serious threat to public health, and its prevalence has been increasing at an alarming rate. The incubation period for the virus is ~1–14 days and all age groups may be susceptible to a fatality rate of about 5.9%. COVID-19 is caused by a novel single-stranded, positive (+) sense RNA beta coronavirus. The development of a vaccine for SARS-CoV-2 is an urgent need worldwide. Immunoinformatics approaches are both cost-effective and convenient, as *in silico* predictions can reduce the number of experiments needed. In this study, with the aid of immunoinformatics tools, we tried to design a multi-epitope vaccine that can be used for the prevention and treatment of COVID-19. The epitopes were computed by using B cells, cytotoxic T lymphocytes (CTL), and helper T lymphocytes (HTL) base on the proteins of SARS-CoV-2. A vaccine was devised by fusing together the B cell, HTL, and CTL epitopes with linkers. To enhance the immunogenicity, the β-defensin (45 mer) amino acid sequence, and pan-HLA DR binding epitopes (13aa) were adjoined to the N-terminal of the vaccine with the help of the EAAAK linker. To enable the intracellular delivery of the modeled vaccine, a TAT sequence (11aa) was appended to C-terminal. Linkers play vital roles in producing an extended conformation (flexibility), protein folding, and separation of functional domains, and therefore, make the protein structure more stable. The secondary and three-dimensional (3D) structure of the final vaccine was then predicted. Furthermore, the complex between the final vaccine and immune receptors (toll-like receptor-3 (TLR-3), major histocompatibility complex (MHC-I), and MHC-II) were evaluated by molecular docking. Lastly, to confirm the expression of the designed vaccine, the mRNA of the vaccine was enhanced with the aid of the Java Codon Adaptation Tool, and the secondary structure was generated from Mfold. Then we performed *in silico* cloning. The final vaccine requires experimental validation to determine its safety and efficacy in controlling SARS-CoV-2 infections.

## Introduction

In December 2019, COVID-19, caused by the severe acute respiratory syndrome coronavirus 2 (SARS-CoV-2) was first discovered in China and has rapidly spread across the world. As of 12:00 noon on June 4, a total of 6,392,319 confirmed cases of COVID-19 have been reported globally, including 383,318 deaths. The prevalence of the disease has been increasing at an alarming rate. There were 1,849,852 cases in the United States, 555,383 in Brazil, 431,715 in Russia, 281,270 in the United Kingdom, and 3,275,736 in a number of other countries (1).

The incubation period for the virus is ~1–14 days, and all age groups are susceptible to a fatality rate of about 5.9%. The most common clinical manifestations are low-grade fever, dry cough, fatigue, and gastrointestinal symptoms (2). About half of all patients with COVID-19 develop shortness of breath, and severe cases may rapidly develop SARS, septic shock, difficult-to-correct metabolic acidosis, and coagulation disorders (3). COVID-19 may also affect other organs, most commonly the heart and kidneys (4–6). Some patients may have mild symptoms, without fever, and may recover after 1–4 weeks (7). Other patients may show signs of serious illness and some may die; however, most patients show favorable progress (8). Male individuals with the disease and aged patients have the worst prognosis. In children, the disease is relatively mild (9).

COVID-19 is caused by a novel single-stranded, positive (+) sense RNA beta coronavirus, which is a pathogen of the *Coronaviridae* family, named SARS-CoV-2 (10). The full-length genome sequences revealed that SARS-CoV-2 has the greatest genetic similarity to bat coronavirus, ~45–90% similarity to severe acute respiratory syndrome-related coronavirus (SARSr-CoV), and a smaller similarity of 20–60% to the Middle East respiratory syndrome-related coronavirus (MERS-CoV) (10). Thus, a bat might be the original host of SARS-CoV-2, but the intermediate host remains undiscovered (10).

The genes of SARS-CoV-2 encode structural proteins and non-structural proteins. Four structural proteins are absolutely vital for viral assembly and invasion of SARS-CoV-2. Spike protein homotrimers constitute the spikes on the viral surface, and these spikes are responsible for attachment to host cells by binding to their receptors (10). The M protein has three transmembrane domains, which determine the shape of the virion, facilitate membrane curvature, and bind to the nucleocapsid. The *E* protein plays an important role in virion assembly and release, as well as involved in viral pathogenesis. The *N* protein has two different domains, both of which bind to the viral RNA genome via totally different mechanisms. In addition, some reports have shown that non-structural proteins are essential for the replication of coronaviruses (10).

Vaccination is a vital tool for the control and elimination of the virus, and the development of a vaccine for SARS-CoV-2 remains an urgent need (11). Traditional methods of vaccine development are time-consuming and very labor-intensive (12). The realm of immunoinformatics tools considers the mechanism of the host immune response to yield additional methodologies in the design of vaccine against diseases are cost-effective and convenient, as *in silico* predictions can reduce the number of experiments needed (13, 14). Dozens of studies have generated epitope-based peptide vaccine of SARS-CoV-2. Baruah and Bose (15) used immunoinformatics tools to discover cytotoxic T lymphocyte (CTL) and B cell epitopes for the spike protein of SARS-CoV-2. Then, Abraham et al. developed a multi-epitope vaccine that was designed using immunoinformatics tools that potentially trigger both CD4^+^ and CD8^+^ T-cell immune responses (16).

Although there are many vaccines generated by immunoinformatics tools, most of these are based on spike protein. The spike protein is responsible for attachment to host cells by binding to angiotensin-converting enzyme 2 (ACE2) (17). A vaccine based on the spike protein could induce antibodies to block SARS-COV binding and fusion or neutralize virus infection (18). But there are still many obstacles, spike protein-based SARS vaccine may induce harmful immune responses that cause liver damage of the vaccinated animals (19). Other virus proteins are considered as the candidates for designing vaccine with protective and less harmful immune responses (20). Vaccine-based on structural and non-structural proteins of the virus is revealed potential vaccine inducing protective immune responses (20, 21). Pandey et al. reported the more scientifically rigorous strategy of multi-epitope subunits based on multiple proteins against parasitic and viral diseases, such as malaria, visceral leishmaniasis, and HIV (22–24). In this present, we employed immunoinformatics to predict multiple immunogenic proteins from the SARS-CoV-2 proteome and thereby design a multi-epitope vaccine. These proteins included non-structural and structural sequences of SARS-CoV-2, their reference sequences were retrieved from the National Center for Biotechnology Information (NCBI) database.

## Materials and Methods

### Retrieving COVID-19 Protein Sequences

The proteins of the SARS-CoV-2 have been reported and reference could get from NCBI (25, 26). The reference sequences of SARS-CoV-2 proteins were retrieved from NCBI Protein Database (https://www.ncbi.nlm.nih.gov/protein) and accession numbers in Table 1, then we stored the reference sequences as a FASTA data type. The proteins with <100 amino acid sequences which are too short to predict epitopes were excluded, the remaining proteins were used for further analysis.

**Table 1:** Details and antigenicity of SARS-CoV-2 proteins.

**No**	**Accession** **number^a^**	**Protein**	**Amino** **acids**	**Antigenic** **value^b^**
1	YP_009724395.1	ORF7a protein	121aa	0.6755
2	YP_009724396.1	ORF8 protein	121aa	0.6392
3	YP_009725305.1	nsp9	113aa	0.6292
4	YP_009725302.1	nsp6	290aa	0.5668
5	YP_009725299.1	nsp3	1945aa	0.5538
6	YP_009725310.1	endoRNAse	346aa	0.5436
7	YP_009724391	ORF3a protein	275aa	0.541
8	YP_009724393.1	Membrane glycoprotein	222aa	0.5184
9	YP_009724397.2	Nucleocapsid phosphoprotein	419aa	0.5133
10	YP_009725295.1	ORF1a polyprotein	4405aa	0.4813
11	YP_009725300.1	nsp4	500aa	0.4759
12	YP_009724390.1	Surface glycoprotein	1273aa	0.4707
13	YP_009724389.1	ORF1ab polyprotein	7096aa	0.4624
14	YP_009725297.1	Leader protein	180aa	0.4497
15	YP_009725309.1	3′-to-5′ exonuclease	527aa	0.4219
16	YP_009725308.1	Helicase	601aa	0.4219
17	YP_009725307.1	RNA-dependent RNA polymerase	932aa	0.4123
18	YP_009725306.1	nsp10	139aa	0.4091
19	YP_009725304.1	nsp8	198aa	0.4063
20	YP_009725298.1	nsp2	638aa	0.4043
21	YP_009725301.1	3C-like proteinase	306aa	0.4037
22	YP_009725311.1	2'-O-ribose	298aa	0.3917
23	YP_009724394.1	ORF6 protein	61aa	0.5719
24	YP_009725296.1	ORF7b protein	43aa	0.5505
25	YP_009725255.1	ORF10 protein	38aa	0.622
26	YP_009725312.1	nsp11	13aa	0.2878
27	YP_009724392.1	Envelope protein	75aa	0.6243

a *The accession number is the National Center for Biotechnology Information (NCBI) reference sequence number*.

b *The antigenic value threshold was > 0.5000*.

### Identifying Antigenicity of Protein Sequences

VaxiJen is the first server for alignment-independent prediction of protective antigens, which overcome the limitations of alignment-dependent methods (27). To identify the potential antigenicity of SARS-CoV-2 proteins, an online prediction server, VaxiJen v2.0 (http://www.ddg-pharmfac.net/vaxijen/VaxiJen/VaxiJen.html) was used to predict the antigenic values of each protein (28). This identification was applied according to the default parameters of the server. Proteins having antigenicity were sorted according to an antigenic score of ≥ 0.5 (Threshold for this model is 0.5) and were selected for further structural modeling (27).

### Structural Modeling of SARS-COV-2 Proteins

There are no available experimental structures of SARS-COV-2 proteins, Phyre 2 provide model regions trough a new ab initio folding simulation with no detectable homology (29). The SARS-CoV-2 proteins were modeled by Phyre 2 server (http://www.sbg.bio.ic.ac.uk/phyre2/). Because the SARS-COV-2 proteins with no detectable homology protein to finish the modeling, we chose the intensive search and output the accurate alignment by the alignment of hidden Markov models.

ModRefiner was used by the GalaxyRefine server (http://galaxy.seoklab.org /cgi-bin/submit.cgi?type=REFINE) (30). The structure assessment was performed by the SWISS-MODEL workspace (https://swissmodel.expasy.org/assess) (31). The three dimensional (3D) models were used for the conformational (discontinuous) B-cell epitope predictions while the sequences were utilized in linear B-cell and T-cell epitope predictions.

### Prediction of CTL Epitopes

NetCTL-1.2 is demonstrated to have a high predictive performance (32). The NetCTL 1.2 server (http://www.cbs.dtu.dk/services/NetCTL/) was applied to predict CTL epitopes for the SARS-CoV-2 at the threshold value of 0.75 with high sensitivity and specificity (32). To cover ~90% of the world's population, three supertypes (A2, A3, and B7) were selected based on artificial neural networks, to predict MHC class I binding epitopes (33). The best candidates for the SARS-CoV-2 vaccine construction were sorted for further prediction, based on a half-maximal inhibitory concentration (IC50) < 500 nm and an integrated score. The IC50 < 500 nm represents epitope has a high affinity to receptor. The integrated score indicated the transporter of antigenic peptides (TAP) transport efficiency, class I binding, and proteasomal cleavage prediction (34–36). Then the specific Treg epitopes were screened and excluded by the EpiToolKit (https://epivax.com/).

### Prediction of Helper T Lymphocyte (HTL) Epitopes

For MHC class II T cell epitope predictions, The Immune Epitope Database server predicted binders based on the percentile rank or MHC binding affinity (37). The Immune Epitope Database server (IEDB; http://tools.iedb.org/mhcii/) was used to predict helper T lymphocyte (HTL) epitopes (37). We chose the combinatorial approach which recommended by IEDB to predict HTL epitopes. The combinatorial approach combined NN-align, SMM-align, CombLib, Sturniolo, and NetMHCIIpan methods (38–42). The 17 alleles of the human leukocyte antigen (HLA) were selected for the prediction at α and β chains, separately (43). For final construction, epitopes were selected based on their scores (low scores indicated favorable binding), the release of interferon-gamma (IFN-γ), induction of emergent properties, and the IC50 < 500 nm.

### Prediction of IFN-γ Inducing Epitopes

The IFN-γ cytokine makes a major contribution to antiviral mechanisms. It excites both native and specific immune responses by activating macrophages and natural killer cells (44). Further, IFN-γ augments the response of MHC to antigens. The IFN-γ epitope server (http://crdd.osdd.net/raghava/ifnepitope/scan.php) was used to recognize IFN-γ epitopes (45). We entered the HTL epitopes with low scores into the IFN-γ epitope server. Positive IFN-γ induction was predicted based on the support vector machine (SVM) hybrid approach. The final HTL epitopes were determined based on IFN-γ induction and MHC Class II binding, both of which facilitate the stimulation of T-helper cells (46).

### Prediction of Line and Conformational B Cell Epitopes

The ABCpred (http://crdd.osdd.net/raghava/abcpred/) and BepiPred linear epitope prediction (http://tools.iedb.org/bcell/result/) servers were utilized to predict linear B cell epitopes. The ABCpred server is based on an artificial neural network (ANN) (47, 48). The linear B cell epitopes of the SARS-CoV-2 protein were predicted at a threshold of 0.5. The BepiPred linear epitope prediction server is based on seven methods: (a) Bepipred-1.0 Linear Epitope Prediction; (b) BepiPred-2.0: Sequential B cell Epitope Predictor; (c) Chou and Fasman beta-turn prediction; (d) Emini surface accessibility scale; (e) Karplus and Schulz flexibility scale; and the (f) Kolaskar and Tongaonkar antigenicity scale (49–54). We used these seven methods separately to predict the average threshold. The overlap between ABCpred and BepiPred severs was selected to determine the candidate epitopes for the SARS-CoV-2 vaccine construction (55).

Unlike T-cell epitopes that are linear continuous stretches of residues, B-cell epitopes are generally conformational (discontinuous) (56). In this study, the ElliPro servers (http://tools.iedb.org/ellipro/) were applied to predict the conformational B-cells epitopes (57). The server predicts epitopes based on PI (Protrusion Index) value. The epitope with PI = 0.9 would include 90% of residues with 10% being outside the ellipsoid, discontinues B-cells epitopes with the top PI value was selected for vaccine designing (57).

### Multi-Epitope Subunit Vaccine Design

To develop the final vaccine, epitopes determined by various immunoinformatics software were linked together with the aid of separate linkers. The CTL epitopes were linked by the AAY linker, HTL epitopes by the GPGPG linker, and B cells were linked by the KK linker (48, 58, 59). To increase the vaccine immunogenicity, the β-defensin (45 mer) amino acid sequence was adjoined to the N-terminal of the vaccine with the help of the EAAAK linker (60). The β-defensin peptides provoke innate immunity cells and recruit naive T cells through the chemokine receptor-6 (CCR-6) (61). The pan-HLA DR binding epitopes (13aa) as well as added to the N-terminal of the vaccine with the aid of the same linker (59). The pan-HLA DR binding epitopes in vaccine construct facilitating binding to many different types of mouse and human MHC-II alleles to induce CD4-helper cell responses (59). To enable the intracellular delivery of the modeled vaccine, a TAT sequence (11aa) was appended to C-terminal (62). Linkers (AYY, KK, and GPGPG) play vital roles in producing an extended conformation (flexibility), protein folding, and separation of functional domains, and therefore, make the protein structure more stable (59).

### Prediction of Allergenicity, Antigenicity

The allergenic proteins induce a harmful immune response, allergenicity of the vaccine should be non-allergic (63). The non-allergic character of the vaccine sequence was evaluated by the AlgPred server (http://www.imtech.res.in/raghava/algpred/) (63). We predicted allergenicity of vaccine sequences choosing a hybrid approach (SVMc+IgE epitope+ARPs BLAST+MAST) with the highest accuracy and sensitivity (63).

The Vaxijen v2.0 server (http://www.ddgpharmfac.net/vaxijen/VaxiJen/VaxiJen.html) was applied to evaluate the antigenicity of the vaccine (27). The antigenicity prediction method was solely based on the physicochemical properties of proteins without recourse to sequence alignment. The precision rate of the server ranged from 70 to 89%.

### Immune Simulations

To determine immune response profile of this multi-epitope vaccine, computational immune simulations were performed by the C-ImmSim online server at (http://kraken.iac.rm.cnr.it/C-IMMSIM/) (64). The C-ImmSim utilizes the Celada-Seiden model for describing both humoral and cellular profiles of a mammalian immune system against designed vaccine. As per the literature, three injections were administrated at different intervals of 1 month. The simulation was performed with default parameters. The vaccine sequence was administered 4 weeks apart. The simulation volume was 1,000, simulation steps was 1,000, random seed was 12,345, and the vaccine injection with no LPS (64).

### Prediction of Various Physicochemical Properties

The ProtParam tool (http://web.expasy.org/protparam/) was used to evaluate the physicochemical properties of the final vaccine protein (65). The physicochemical properties included the number of amino acids, molecular weight, theoretical isoelectric point (pI), amino acid composition, atomic composition, formula, extinction coefficients, estimated half-life, instability index, aliphatic index, and grand average of hydropathicity (GRAVY) (66). The molecular weight and theoretical pI were computed by user-entered sequences. The amino acid and atomic compositions were self-explanatory. The extinction coefficient of a protein was based on information about its amino acid composition. The instability index of a protein indirectly indicated the stability of the protein. If the computed instability index of protein was <40, it was regarded as a stable protein, while values >40 were regarded as unstable. *In vivo* half-life evaluation of proteins was based on the principle of the “N-end rule.” Furthermore, GRAVY is a measurement of the hydrophobic nature of the protein, which is calculated by determining the total hydropathy of all amino acids divided by the number of amino acid residues in the protein.

To avoid inducing pathogenic priming and autoimmunity, the sequence homology of the final vaccine to human protein was screened by BLASTp online server (https://blast.ncbi.nlm.nih.gov/Blast.cgi) (67). An ideal vaccine should have non-sequence to human proteins.

### Prediction, Refinement, and Quality Assessment of the Tertiary Structure of the Developed Vaccine Construct

The designed vaccine was a reconstructed protein with no detectable homology (29). Phyre2 incorporates an ab initio folding simulation to model regions of proteins with no detectable homology. The Phyre 2 server (http://www.sbg.bio.ic.ac. uk/phyre2/) was used to predict the three-dimensional structure of the designed vaccine. The server generates a full-length 3D model of a protein sequence by employing both multiple template modeling and simplified ab initio folding simulation (29).

To enhance the overall and partial structural quality of the protein, the output 3D structure of the final vaccine from the Phyre 2 server was further refined by the GalaxyRefine server (http://galaxy.seoklab.org/cgi-bin/submit.cgi?type=REFINE) (30). The GalaxyRefine server predicted five refined models of our developed vaccine construct, in which Model 1 was made by the structural perturbation based simply on the clusters of the side chains; whereas, Models 2–5 were generated by deeper perturbations of loops and secondary structural elements (30).

For the assessment of the tertiary structure of the final vaccine protein, a Ramachandran plot was performed by the SWISS-MODEL workspace (https://swissmodel.expasy.org/assess) (31). The Ramachandran plot illuminates favored regions for backbone dihedral angles against amino acid residues in protein structure (31). The Structure Assessment page shows the most relevant scores provided by Molprobity and help we easily identify where residues of low quality lie in their model or structure (31). Then, ProSA-web (https://prosa.services.came.sbg.ac.at/prosa.php) was employed in the final vaccine protein structure validation. A positive *Z*-score commonly means an erroneous or erratic section found in the generated 3D protein model (68).

### Molecular Docking of the SARS-CoV-2 Vaccine Construct With the Related Antigenic Recognition Receptor

To revealing the binding affinity between the vaccine construct and antigenic recognition receptors of toll-like receptor-3 (TLR3, 2A0Z) and major histocompatibility complex (MHC-I, 4WUU, and MHC-II, 3C5J) present on the surface of immune cells (69). Docking analysis was performed using the ClusPro server (https://cluspro.bu.edu/login.php?redir/queue.php). TLR3 act as receptors for antigenic recognition. The ClusPro server computed the models based on electrostatic interactions and desolvation energy (69). To reconfirm the binding affinity of the designed vaccine construct between these receptors, the PatchDock server (https://bioinfo3d.cs.tau.ac.il/PatchDock/) was used for docking (70). The server predicted the potential complex with the help of three algorithm-molecular shape representations, surface patch matching, filtering, and scoring (70). After the acquisition of the output from the PatchDock server, the complexes were refined by the FireDock algorithm, which predicted the optimal complex with the aid of energy functions (70).

### Molecular Dynamic Simulation

The pdb file of vaccine protein and receptor complex (TLR3, MHC-I, and MHC-II) were used to start the molecular dynamic (MD) simulations. The complexes were placed in a octahedron box of water molecules represented by the three-point charge SPC model, whose boundary is at least 10 Å from any protein atoms. The solvated protein was subsequently neutralized by chloridions. Covalent bonds involving hydrogen atoms were constrained using the LINCS algorithm, and long-range electrostatic interactions were treated with particle-mesh Ewald employing a real-space cutoff of 10 Å. The system was first briefly minimized with backbone atoms restrained to the initial coordinates to remove close contacts, and the restrained system was gradually heated to 300 K under constant volume conditions in 100 ps. Each system was equilibrated for 1 ns using the constant isothermal-isobaric ensemble at 1 atm and 300 K without any restraints. The Parrinello-Rahman barostat and a V-rescale thermostat were used with an integration time step of 2 fs. Production run MD simulations were performed for 10 ns with coordinates recorded every 10 ps. All simulations were performed using GROMACS 2018.2 along with the GROMOS96 54a7 force field (16, 24).

### Codon Adaptation and *in silico* Cloning

**Figure 1:**
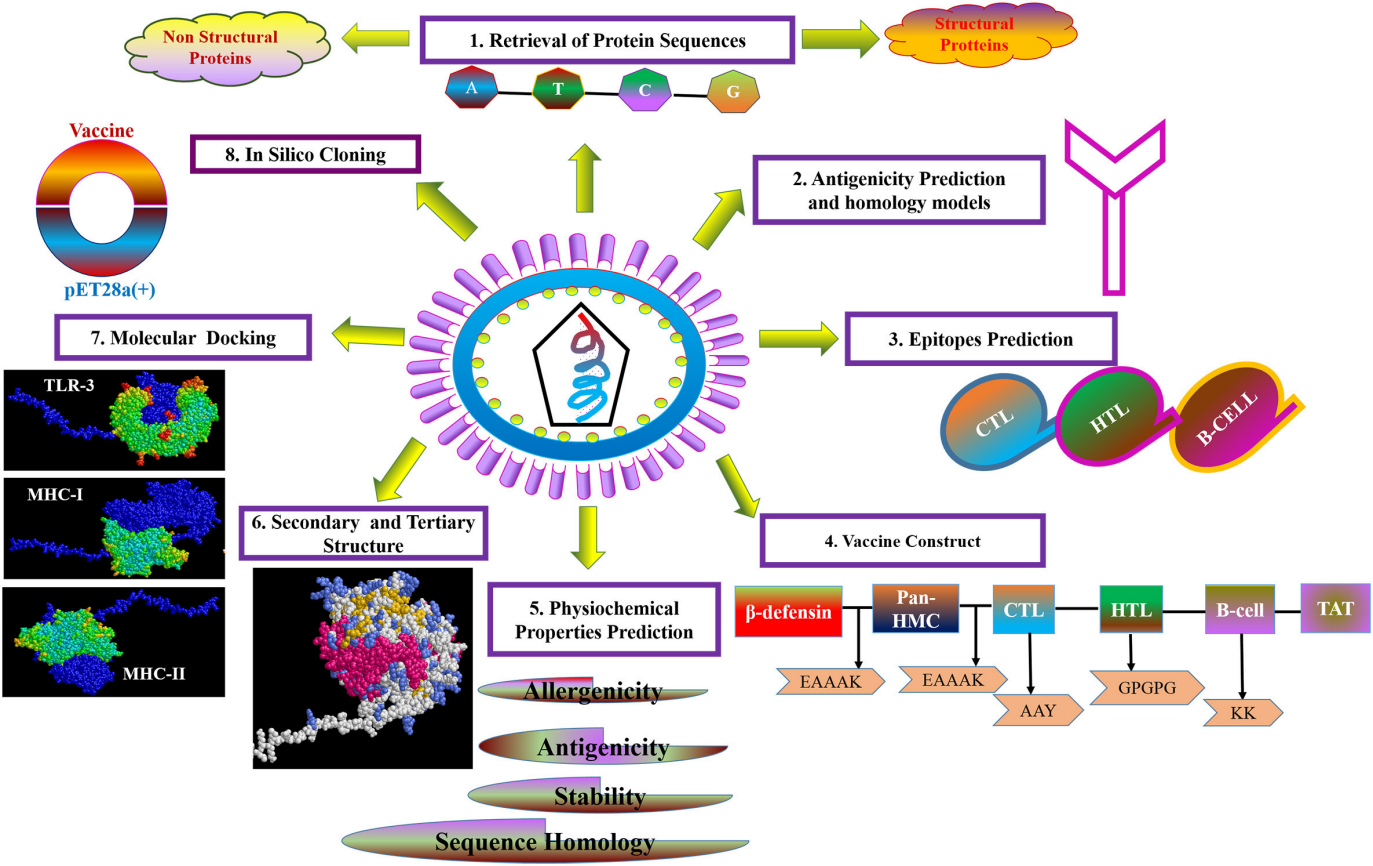
Flow diagram of design strategy, representing the steps of the construct of the multi-epitope subunit vaccine.

For the purpose of cloning, codon adaptation of the designed vaccine was performed for analyzing the codon usage by the prokaryotic organism (*Escherichia coli, E. coli*). The Java Codon Adaptation tool (http://www.jcat.de/) was used to optimize codon (71). Then the secondary structure of mRNA was predicted by Mfold (http://unafold.rna.albany.edu/?q=mfold) (72). For raising the expression efficiency of the final vaccine protein, the *E. coli* K12 strain was chosen. For the valid translation of the vaccine gene, we proofread and avoided rho-independent transcription termination, prokaryote ribosome binding site, and cleavage site of restriction enzymes. Restriction endonuclease sites XhoI and BamHI were appended to *N* and *C* terminals of vaccine, respectively. Then, it was inserted into the pET28a (+) vector between the XhoI and BamHI. The flow chart of the designed work is shown in Figure 1.

## Results

The strategy of vaccine construction is presented in Figure 1.

### Antigenicity Analysis of SARS-CoV-2 and Selection of Protein Sequences for Vaccine Construction

The proteome of SARS-CoV-2 was retrieved, which comprised 27 proteins. The reference sequences of those proteins were retrieved in the FASTA format and their details are presented in Table 1. Five proteins with <100 amino acid sequences are too short to predict epitopes (ORF6 protein, ORF10 protein, ORF7b protein, nsp11, and envelope protein) were excluded.

In order to develop a subunit vaccine, it is critical to identify candidate proteins that are important for inducing a protective immune response (27). The remaining 22 proteins sequence were relayed to the VaxiJen server to determine their antigenicity based on the antigenic scores (Table 1). Proteins with antigenic scores >0.5 were selected for further analysis (28). Nine proteins, namely ORF7a protein, ORF8 protein, nsp9, nsp6, nsp3, endoRNAse, ORF3a protein, membrane glycoprotein, and nucleocapsid phosphoprotein were finally selected for further epitope prediction.

There is no available experimental structures of these nine proteins, we predicted homology models for the nine proteins applying the normal mode of phyre2 online server. The most suitable templates for the nine proteins were identified to be the PBD entries (Table S1). All of the modeled structures were showed over 90% residues in the Ramachandran favored region Figure S1 and Table S2.

### Identification of Cytotoxic T Cell Epitopes

The prediction of CTL epitopes (9 mer) was performed by the NetCTL server. The binder sites were determined based on three supertypes (A2, A3, and B7), with a 95% coverage rate of the world's population. Nine proteins were selected based on antigenicity. One epitope of each supertype was selected based on the highest score and an IC50 value < 500 nm. Then the specific Treg-inducing epitopes were excluded by Epitoolkit. A total of 18 epitopes were selected from nine proteins as the candidates for the construction of the vaccine (Table 2).

**Table 2:** Predicted cytotoxic T lymphocyte (CTL) epitopes of SARS-CoV-2 proteins utilized for the construction of a multi-epitope subunit vaccine.

**Protein**	**CTL epitopes predicted using the NetCTL server**		
	**A2 supertype (IC50)**	**A3 supertype (IC50)**	**B7 Supertype (IC50)**
ORF7 protein	KLFIRQEEV (58.81)	TLCFTLKRK (219.46)	SPIFLIVAA (231.29)
nsp9	ALLSDLQDL (8.28)		
nsp6	FLLPSLATV (2.7)	SAFAMMFVK (92.76)	MPASWVMRI (171.46)
nsp3		VMYMGTLSY (72.50)	
endoRNAse	LLLDDFVEI (21.12)		SPFGHSLTL (10.75)
ORF3a protein		IMRLWLCWK (98.03)	IPIQASLPF (13.56)
Membrane glycoprotein	GLMWLSYFI (11.32)	LSYFIASFR (138.74)	LPKEITVAT (244.01)
Nucleocapsid phosphoprotein	LLLDRLNQL (84.26)	KTFPPTEPK (69.08)	FPRGQGVPI (3.82)

*The half-maximal inhibitory concentration (IC50) value was > 500 nm, which ensured a higher binding capability of the selected epitopes to MHC molecules*.

### Identification of Helper T Lymphocyte Epitopes

The HTL epitopes (15 mer) were evaluated for three HLA supertypes: HLA-DR (DRB1^*^01:01, DRB1^*^07:01, DRB1^*^09:01, DRB3^*^01:01, DRB4^*^01:01); HLA-DQ (DQA1^*^01:01/DQB1^*^05:01, DQA1^*^01:02/DQB1^*^06:02, DQA1^*^03:01/DQB1^*^0:02, DQA1^*^04:01/DQB1^*^04:02, DQA1^*^05:01/DQB1^*^02:01, DQA1^*^05:01/DQB1^*^03:0 1); and HLA-DP (DPA1^*^01/DPB1^*^04:01, DPA1^*^01:03/DPB1^*^02:01, DPA1^*^02:01 /DPB1^*^01:01, DPA1^*^02:01/DPB1^*^05:01, DPA1^*^03:01/DPB1^*^04:02). We sorted the top epitopes with the lowest scores (low scores indicated the highest binding capability) from three supertypes. The best candidate was then selected based on positive IFN-γ induction and an IC50 < 500 nm. Then the specific Treg-inducing epitopes were excluded by Epitoolkit. Thus, a total of 14 epitopes were selected for vaccine design (Table 3).

**Table 3:** Predicted Helper T lymphocyte (HTL) epitopes of SARS-CoV-2 proteins utilized for the construction of a multi-epitope subunit vaccine.

**Epitope**	**Allele (score)**	**IC50**
**ORF8 protein**		
HFYSKWYIRVGARKS	HLA-DRB1*07:01 (0.06) HLA-DRB1*01:01 (0.07)	32.2 39.5
DFLEYHDVRVVLDFI	HLA-DQA1*05:01/DQB1*02:01 (0.01) HLA-DQA1*01:01/DQB1*05:01 (0.5)	276 101.9
IHFYSKWYIRVGARK	HLA-DPA1*02:01/DPB1*01:01 (0.01) HLA-DPA1*03:01/DPB1*04:02 (0.02) HLA-DPA1*01:03/DPB1*02:01 (0.09)	281.8 42.8 79.4
**nsp9**		
KGLNNLNRGMVLGSL	HLA-DQA1*05:01/DQB1*03:01 (0.04) HLA-DQA1*01:02/DQB1*06:02 (0.62)	62 95
GPKVKYLYFIKGLNN	HLA-DPA1*01:03/DPB1*02:01 (0.01) HLA-DPA1*02:01/DPB1*05:01 (0.02) HLA-DPA1*02:01/DPB1*01:01 (0.52)	61 79.7 109.3
**nsp3**		
TAFGLVAEWFLAYIL	HLA-DQA1*05:01/DQB1*02:01 (0.01) HLA-DQA1*01:01/DQB1*05:01 (0.14)	50 46.4
AAIMQLFFSYFAVHF	HLA-DPA1*01:03/DPB1*02:01 (0.01) HLA-DPA1*01/DPB1*04:01 (0.01) HLA-DPA1*02:01/DPB1*01:01 (0.02)	8.7 78.5 110.8
**endoRNAse**		
MEIDFLELAMDEFIE	HLA-DQA1*03:01/DQB1*03:02 (0.01) HLA-DQA1*05:01/DQB1*02:01 (0.03) HLA-DQA1*01:01/DQB1*05:01 (0.07)	97.6 12.9 37.9
GLAKRFKESPFELED	HLA-DPA1*01/DPB1*04:01 (0.02) HLA-DPA1*02:01/DPB1*05:01 (0.12)	108.6 453
**ORF3a**		
ACFVLAAVYRINWIT	HLA-DRB1*07:01 (0.01) HLA-DRB1*01:01 (0.02)	19.9 12.4
**membrane glycoprotein**		
ACFVLAAVYRINWIT	HLA-DQA1*05:01/DQB1*02:01	321.4
KLIFLWLLWPVTLAC	HLA-DPA1*03:01/DPB1*04:02 (0.01) HLA-DPA1*02:01/DPB1*01:01 (0.52) HLA-DPA1*01:03/DPB1*02:01 (0.75)	187.6 133.8 21.3
DDQIGYYRRATRRIR	HLA-DRB1*01:01 (0.01) HLA-DRB1*07:01 (0.01) HLA-DRB3*01:01 (0.01)	223 43 79.2
**nucleocapsid phosphoprotein**		
GKMKDLSPRWYFYYL	HLA-DPA1*01:03/DPB1*02:01 (0.08)	194.7

*The half-maximal inhibitory concentration (IC50) value was < 500 nm, which ensured a higher binding capability of selected epitopes to MHC molecules*.

### Identification of Line and Conformational B-Cell Epitopes

We used the ABCpred and BepiPred servers to identify the line B cell candidate epitopes. All predicted epitopes from both servers were compared, and only the overlapping epitopes were selected for the development of the vaccine. The line epitopes identified by ABCpred had prediction scores ranging from 0.52 to 0.93, and line epitopes identified by BepiPred had prediction scores ranging from 0.5 to 1. Among these line epitopes, only 12 (16 mer) were found to be common or partly common in both servers (Table 4). These 12 line epitopes were selected for vaccine construction (Table 4).

**Table 4:** Predicted line B cell (BCL) epitopes of SARS-CoV-2 proteins utilized for construction of a multi-epitope subunit vaccine.

**Protein**	**Sequence**	**Start position**	**Score**
ORF7 protein	SGTYEGNSPFHPLADN	37	0.92
ORF8 protein	KSPIQYIDIGNYTVSC	68	0.88
	HFYSKWYIRVGARKSA	40	0.87
nsp9	KGPKVKYLYFIKGLNN	81	0.93
	AGTTQTACTDDNALAY	16	0.91
endoRNAse	DFLELAMDEFIERYKL	212	0.76
ORF3a protein	TSPISEHDYQIGGYTE	176	0.93
Membrane	HVQIHTIDGSSGVVNP	243	0.91
glycoprotein	YRIGNYKLNTDHSSS	199	0.69
	NGTITVEELKKLLE	5	0.61
Nucleocapsid phosphoprotein	KSAAEASKKPRQKRTA	249	0.93
	EGALNTPKDHIGTRNP	136	0.93

The non-continuous B cell epitopes were predicted by the ElliPro severs, a total number of 27 non-continuous B cell epitopes were generated from ElliPro. Amino acid residues, sequence location, the number of residues, and the PI scores of the predicted conformational epitopes are shown in Table 5 and the graphical depiction of these epitopes can be seen in Figure S2. Twenty-four epitopes were excluded because it added the allergenicity of vaccine, three epitopes were marked red and selected for vaccine construction.

**Table 5:** Predicted conformational B cell (BCL) epitopes of SARS-CoV-2 proteins.

**R Residues NO. score**			
**ORF7a protein**			
1	R25, G26, T27, T28, L30, K32, E33, P34, C35, S36, S37, G38, P45, H47, P48, L49, A50, D51, N52, K53, C58, C67, P68, D69, G70, V71, R80, S81, V82, S83, P84, K85, L86, F87, I88, R89, E91, E92, E95, L96	40	0.675
**ORF8 protein**			
2	C37, P38, I39	3	0.558
3	Q23, S24, C25, T26, Q27, H28, Q29, P30	8	0.556
**nsp9**			
4	K58, S59, D60, G61, T62, G63, T64	7	0.831
5	D47, V76, D78, T79, P80, K81, G82, P83, K84, V85, G104, A107, A108, T109, V110, R111,	17	0.729
6	N1, N2, E3, L4, S5, P6, V7, A8, L9, T34, T35, K36, G37, G38, E70, K92, G93, L94, N95, N96, L97	21	0.659
7	T18, T19, Q20, T21, A22, C23, T24, D25, L48, Q49, D50, L51	12	0.647
**nsp6**			
8	G258, L259, L260, P261	4	0.786
9	L275, L276, G277, V278, G279, G280, K281, P282, C283, I284	10	0.641
**nsp3**			
10	S675, S676, K677, T678, P679, E680, E681, H682, F683, I684, E685, T686, I687, S688, L689, A690, G691, S692, Y693, K694, D695, W696, S697, Y698, S699, G700, Q701, S702, T703, Q704, L705, G706, I707, E708, F709, L710, K711, R712, G713, D714, K715, S716, V717, Y718, Y719, T720, S721, N722, P723, T724, T725, F726, H727, L728, D729, G730, E731, V732, I733, T734, F735, D736, N737, L738, L741, R745	66	0.818
11	N922, L923, D924, S925, C926, K927, R928, V929, L930, N931, V932, V933, C934, K935, T936, C937, G938, Q939, Q940, Q941, T942, T943, L944, K945, G946, K962, K963, G964, V965, Q966, I967, P968, C969,T970, C971, G972, K973, Q974, A975, T976, K977, Y978, L979, V980, Q981, Q982, E983, S984, P985, F986	50	0.719
12	K839, P841, Q842, V843, N844, G845, L846, T847, W851, A852, D853, N854,N855, C856, L956, S957, A991, P992, P993, A994, Q995, Y996, E997, L998, K999, H1000, G1001, T1002, F1003, T1004, E1008, Y1009, T1010, G1011, N1012, Y1013, Q1014, C1015, G1016, H1017, K1019, T1022, S1023, K1024, E1025, T1026, L1027, Y1028, C1029, I1030, D1031, G1032, A1033, L1034, L1035, T1036, K1037, S1038, S1039, E1040, Y1041, K1042, G1043, P1044, I1045	65	0.648
13	D806, D807, T808, L809, V811, E812, F814	7	0.62
14	K1051, E1052, N1053	3	0.602
**endoRNAse**			
15	S1, L2, E3, N4, V5, A6, F7, N8, V9, V10, N11, K12, G13, H14, F15, D16, G17, Q18, Q19, G20, E21, V22, P23, V24, S25, I26, I27, N28, N29, T30, V31, Y32, T33, K34, V35, D36, G37, V38, D39, V40, E41, L42, E44, N45, K46, T47, T48, L49, P50, V51, N52	51	0.755
16	E145, G146, S147, V148, K149, G150, L151, G169, E170, A171, V172, K173	12	0.707
17	L200, P205, S207, M209, I211, D212, L214, E215, L216, A217, M218, D219, E220, F221, I222, E223, R224, Y225, L227, E228, G229, Y230, A231, F232, E233, H234, I235, Y237, G238, D239, F240, S241, H242, S243, Q244, L245, G246, K256, R257, F258, K259, E260, S261, P262, E264, F279, T281, D282, A283, Q284, T285, G286, S287, S288, K289, C290, K307, S308, Q309, D310, L311, S312, V313, V314, S315, K316, V317, M330, L331, W332, C333, K334, D335, G336, H337, V338, E339	77	0.699
18	T98, I99, G100, C102, S103, M104, T105, D106, I107, A108, K109, K110, P111, T112, E113, T114, I115, C116, A117, P118, L119, T120, G125, R126, V127, D128, G129, V131, D132, L133, F134, R135, N136, A137, R138, N139, K181, V182, D183, G184, V185, V186, Q187	45	0.655
19	Q152, P153, S154	3	0.581
**ORF3a**			
20	H78, C81, N82, L83, L84, L85, L86, F87	8	0.69
21	V97, A98, A99, G100, L101, E102, F105, Y109	8	0.668
22	Q70, L71, K75	3	0.613
**Membrane glycoprotein**			
23	N21, L22, V23, I24	4	0.731
24	N5, G6, T7, I8, T9, V10, E11, K	8	0.588
**Nucleocapsid phosphoprotein**			
25	K338, L339, D340, D341, K342, D343, P344, N345, F346, K347, D348, V350, I351, N354, I357	15	0.747
26	G316, M317, S318, R319, I320, G321, M322, E323, V324, T325, P326, S327, G328, T329, W330, L331, G335	17	0.689
27	A252, E253, A254, S255, K256, K257, P258, K261, R262, A264, T265, K266, A267, Y268, N269, Q272, G278, P279, E280, T282, Q283, N285, G287, D288, Q289, E290, R293, Q294, D297, Y298, K299, H300, D358, A359, Y360, K361, T362, F363, P364	36	0.567

### Construction of the Subunit Vaccine

The best candidate epitopes were used for the construction of the vaccine. A total of 18 CTL epitopes, 14 HTL epitopes, 12 linear, and three non-continuous B cell epitopes were fused together with the aid of linker sequences. The CTL epitopes were linked by AYY (The AAY liner helps the epitopes produce suitable sites for binding to TAP transporter and enhances epitope presentation), the HTL epitopes were combined with the aid of GPGPG (The GPGPG linker stimulate HTL responses and conserve conformational dependent immunogenicity of helpers as well as antibody epitopes), and B cell epitopes were merged with the aid of KK. The final to enhance vaccine immunogenicity, the human β-defensin-3 sequence (45aa) and pan-HLA DR binding epitopes (The pan-HLA DR binding epitopes in vaccine construct facilitating binding to many different types of mouse and human MHC-II alleles to induce CD4-helper cell responses.) was added to the N-terminal of the vaccine with the aid of the EAAK linker. To enable the intracellular delivery of the modeled vaccine, a TAT sequence (11aa) was appended to C-terminal. The vaccine was developed to be 864 amino acids in length (Figure S3). The sequence homology of final vaccine protein to human protein sequence shown that there were no significant alignments (Figure S4).

### Evaluation of Allergenicity, Antigenicity, and Physiochemical Parameters of the Vaccine

The allergenic character of the vaccine was determined by the AlgPred server and was based on the hybrid approach (SVMc + IgE epitope + ARPs BLAST + MAST) with a 93.5% coverage. The vaccine was non-allergen with 84% accuracy and 82.78% sensitivity at threshold value was −0.2. Similarly, the antigenic nature of the vaccine construct was evaluated and showed that the protein was a favorable antigen with a global prediction score of a protective antigen of 0.5308 (Probable antigen). The default threshold value for antigenicity was 0.4 in the virus model.

Moreover, the vaccine constructs contained 864 amino acids, and its molecular weight was 95.4 kDa. The theoretical pI was predicted to be 9.71. The vaccine contained 63 negatively charged residues and 125 positively charged residues. The vaccine construct was composed of 13,541 atoms, and its chemical formula was C_4395_H_6791_N_1153_O_1174_S_28_. The computed instability index was 32.84, which was <40, classifying the vaccine as a stable protein. The estimated half-life was 1 h *in vitro*. *In vivo*, the estimated half-lives in yeast and *Escherichia coli* are greater 30 min and 10 h, respectively. The aliphatic index of the vaccine construct was 79.29, which suggests a high thermostability. The GRAVY value of the vaccine construct was −0.215, which indicated the hydrophobicity of the protein.

### The Immune Response Profile *in silico* Immune Simulation

**Figure 2:**
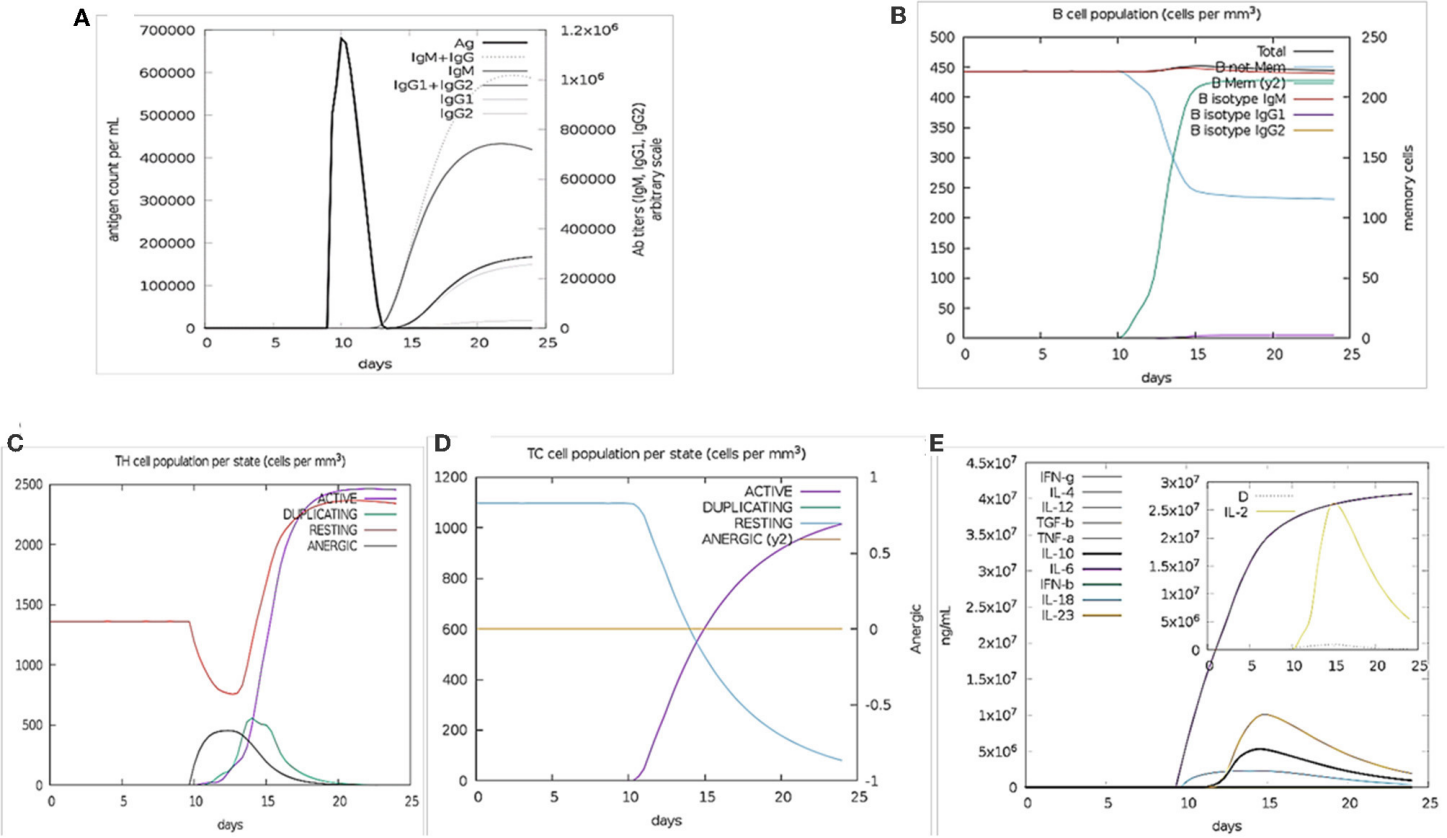
Immune Simulation results by C-ImmSim. **(A)** The immunoglobulins production represent proliferation of immune response after the vaccine administration. Various subtypes of immunoglobulin are represented as colored peaks. **(B)** The active B-cell population is observed with the administration of vaccine. **(C)** The generation of Helper-T cells. **(D)** The generation of cyototoxic-T cells were found after the vaccine injection. The RESTING indicates to the cells, which were not shown to the antigens while ANERGIC indicates the tolerance level of antigen. **(E)** The cytokine profile shows that the induced IFN-γlevel upon administration of vaccine. The inset graph indicating the Simpson Index, D of IL- 2. Simpson Index, D was inferred as the measurement of diversity.

The immune stimulation of the final vaccine was performed using C-ImmSim online server, which gives the immune profiles of the designed vaccine. The proliferation in the secondary and tertian immune response were identified by IgG1 + IgG2 and IgM, as well as, the decreasing of the antigen count IgG + IgM showed the proliferated (Figure 2A). The stimulation result revealed the development of immune response after immunization. B cell population was highly stimulated upon immunization (Figure 2B). Similarly, the cytotoxic and helper T cell levels were proliferated that suggested the development of secondary and tertian immune response (Figures 2C,D). During the exposure time, it was also observed that the production of IFN-γafter immunization (Figure 2E). These results were significant for the immune response against SARS-CoV-2. Hence,

### Prediction, Refinement, and Quality Assessment of the Tertiary Structure of the Developed Vaccine Construct

The tertiary structure of the full-length vaccine sequence was predicted by Phyre 2, and it was applied for refinement and further analysis. Twenty-five templates were employing modeling as Figure S5 shown. There were three templates from human defensin which were we added in to enhance the immunogenicity, others from virus (Figure S5). The immune epitopes were not structural homology to human proteins that could avoid inducing autoimmune. The secondary structure of the predicted model contained 18% alpha-helix, 21%TM helices 44% beta-sheets, and 27% disordered Figure S6.

**Figure 3:**
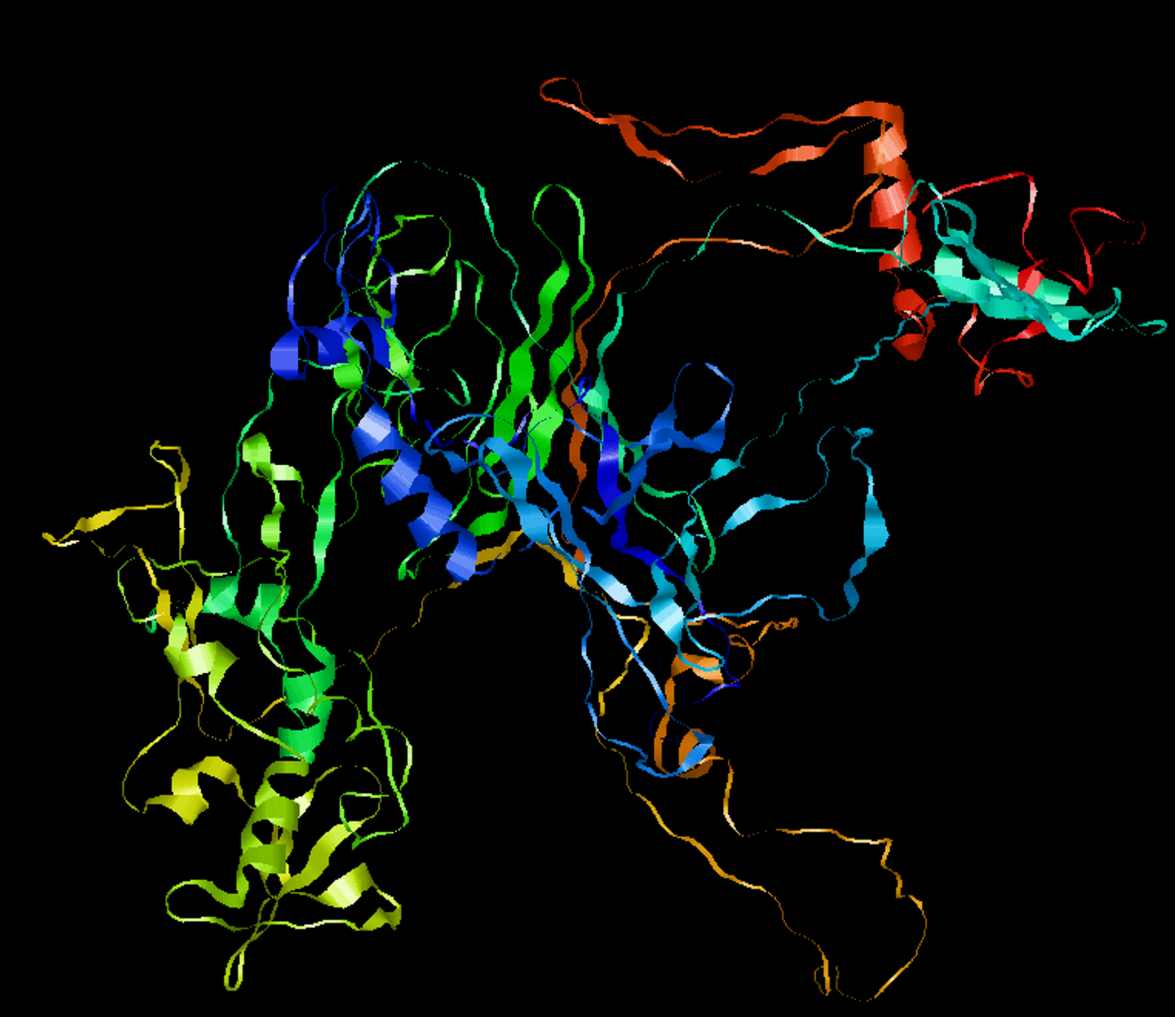
Refinement of the SARS-CoV-2 vaccine construct. Representative 3D image of the tertiary structure of the 2019nCOV vaccine after modeling.

**Figure 4:**
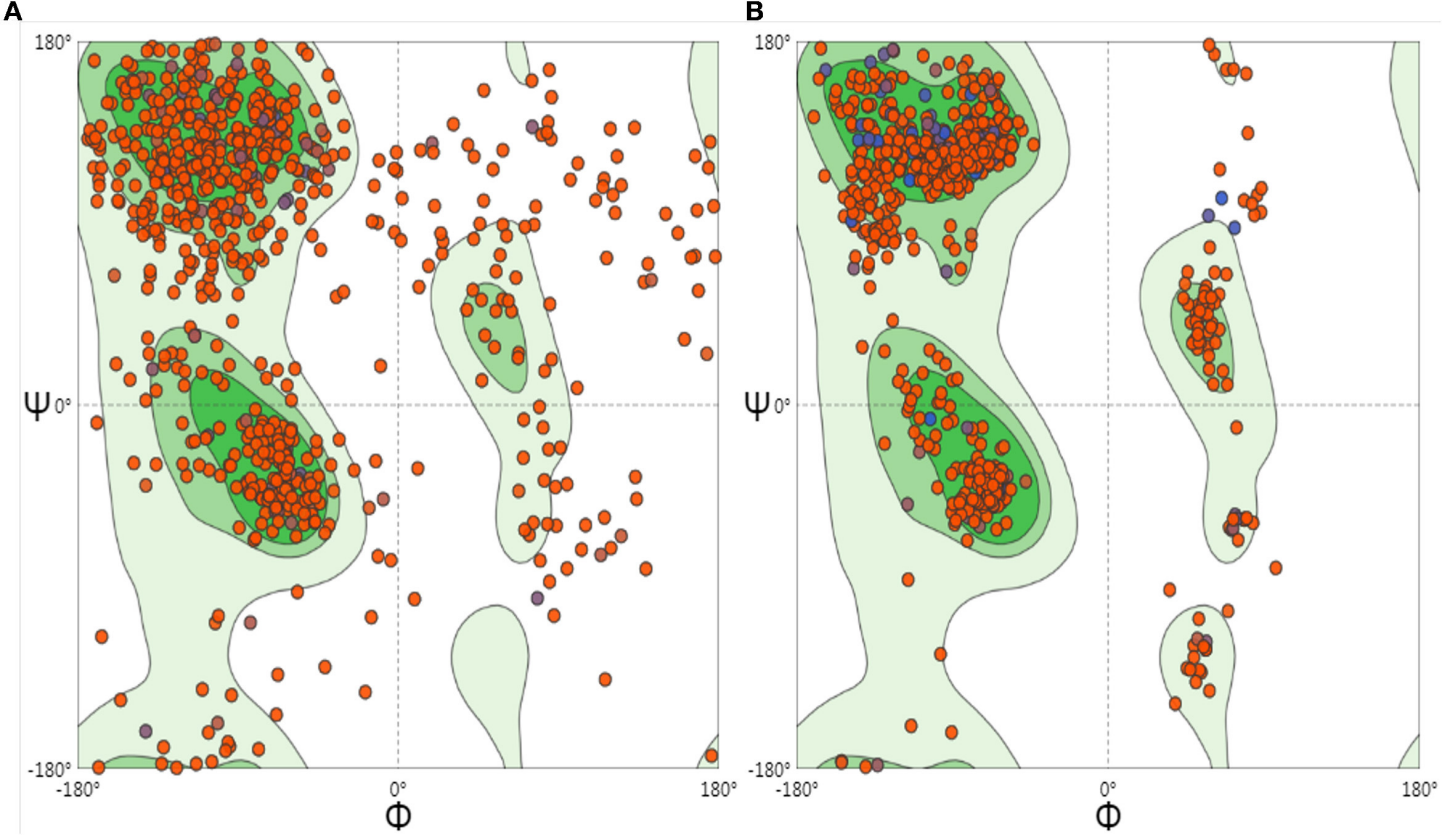
Ramachandran plots to initiate and refine the 3D structure of the vaccine construct illustrated using the SWISS-MODEL/Structure Assessment. **(A)** Shows the Ramachandran plot of initiate model, **(B)** shows the Ramachandran plot of refining the model.

To optimize the 3D structure of the modeled protein, the initial model was refined in the GalaxyRefine server. The GalaxyRefine server-generated five models based on the root-mean-square deviation (RMSD) and MolProbity algorithm. The details of the five models are shown in Table S3. Model 1 with the top Ramachandran favored, therefore selected for docking purposes (Figure 3). A model with more residues in the Ramachandran favored region, less in outliers region and rotamer region was considered as a more ideal one. The initial model generated from Phyre 2 server and refine model from GalaxyRefine were evaluated with the aid of the SWISS-MODEL workspace. The initial model was 63.46% of residues in the Ramachandran favored region, 19.49% in the Ramachandran outliers region, and only 10.22% in the rotamer region (Figure 4). The refine model was 89.1% of residues in the Ramachandran favored region, 2.09% in the Ramachandran outliers region, and only 0.15% in the rotamer region (Figure 3). Other favorable parameters of the refined model were as follows: GDT score of 0.9922, RMSD value of 0.260, MolProbability of 2.049, clash score of 8.9, and poor rotamers totaling 0.3 (Table S1).

**Figure 5:**
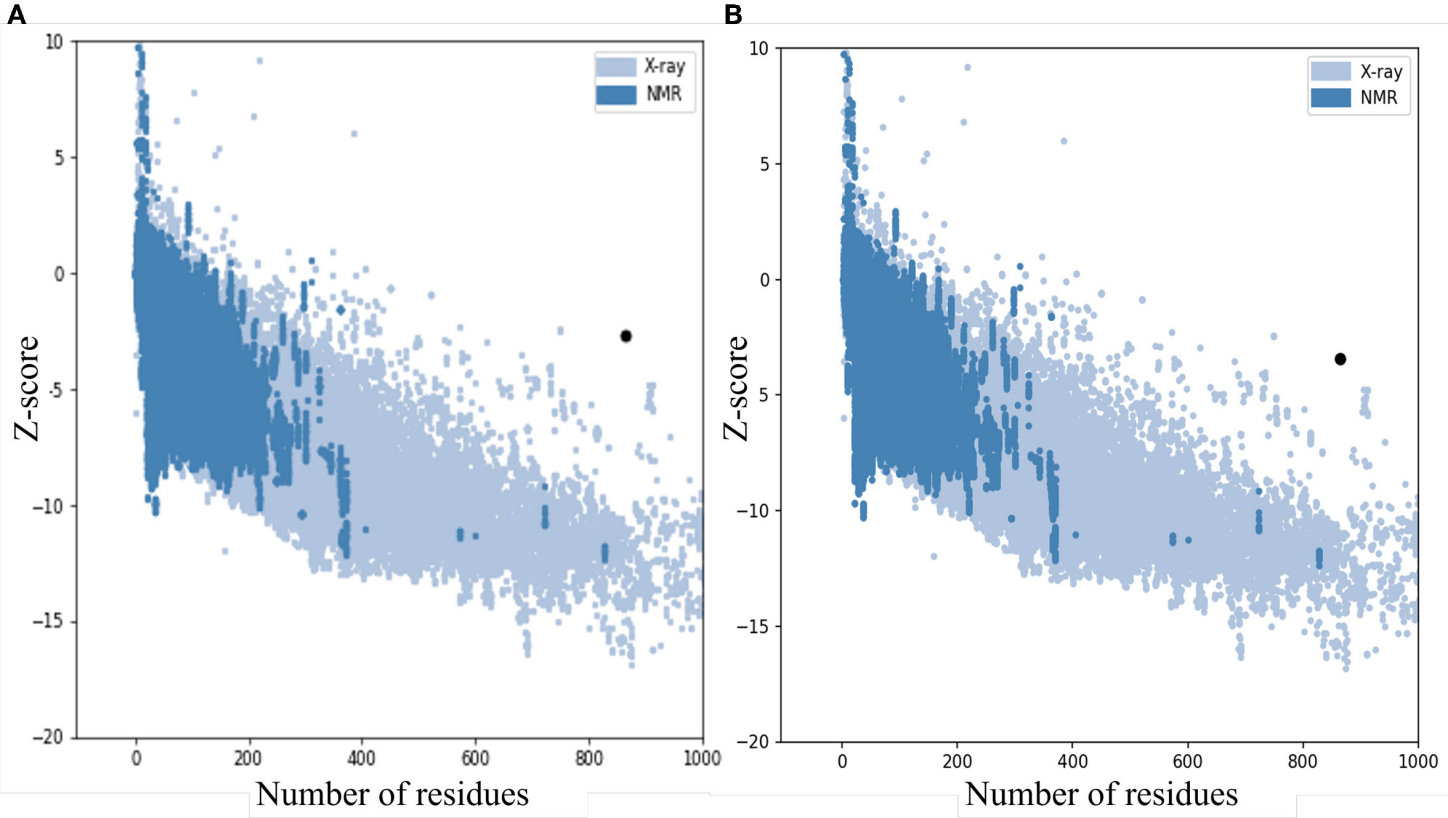
*Z*-Score plot for the 3D structure of the final vaccine. The *Z*-score of **(A)** the initial model is 2.81 and **(B)** The *z*-score of the refined model is 3.64, both of two models not in the range of native protein conformation. *Z*-Score plot contains *z*-scores of all experimental protein chains in PDB determined by NMR spectroscopy (dark blue) and X-ray crystallography (light blue).

The quality and potential errors in the final vaccine 3D model were verified by ProSA-web. The *Z*-score indicates overall model quality, the model with a lower *Z*-score was considered as the higher quality one. The *z*-score of the initial model was −2.81, refine model is −3.64 (Figure 5).

### Molecular Docking of Final Vaccine Construct With the Relatively Antigenic Receptor

**Figure 6:**
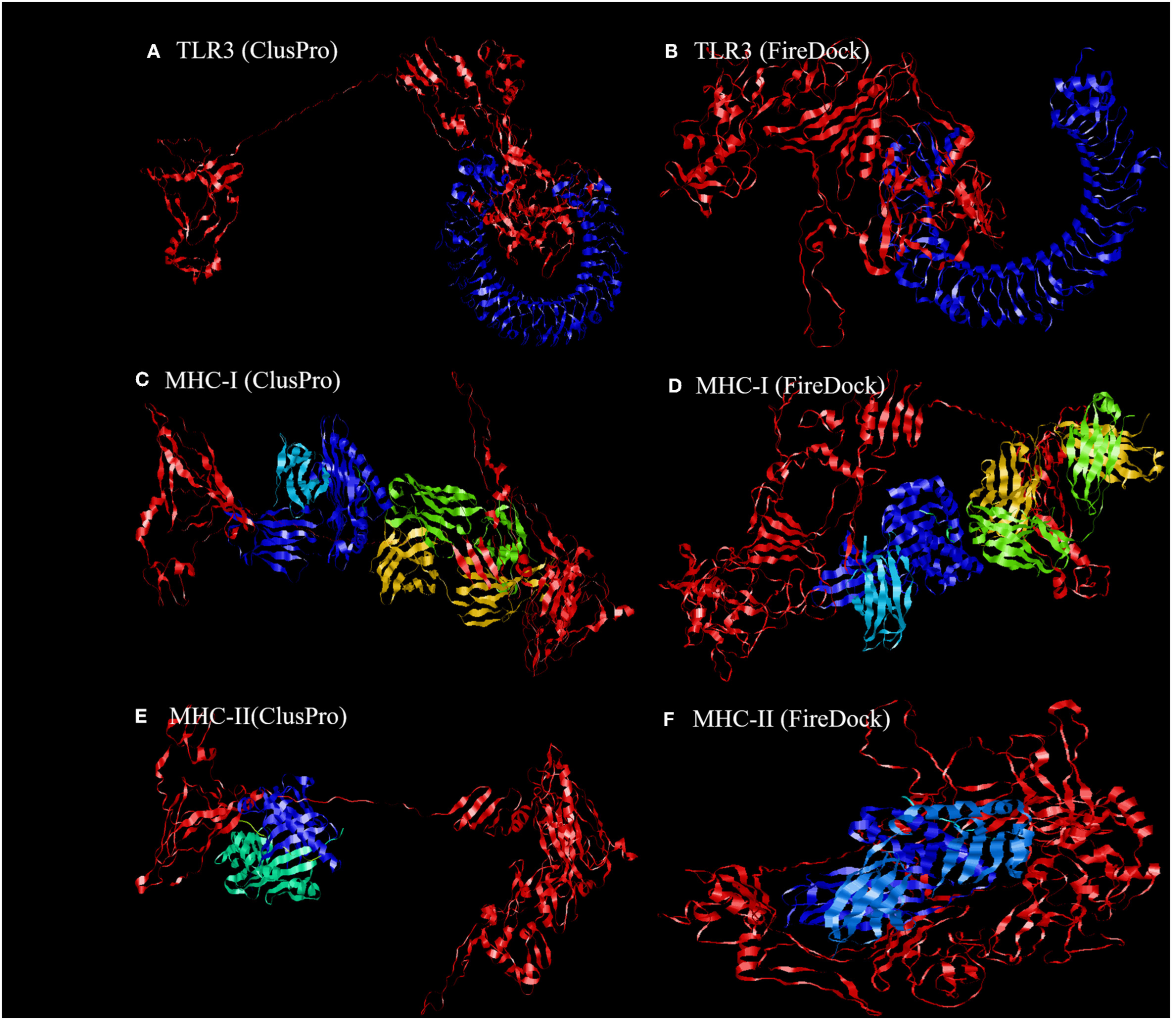
Representation of the ligand-receptor docked complex. **(A,C,E)** show the molecular docking of the vaccine construct (red color) and TLR-3, MHC-I, and MHC-II receptors (other colors) illustrated using the ClusPro software. **(B,D,F)** show the molecular docking of the vaccine construct (red color) and TLR-3, MHC-I, and MHC-II receptors (other colors) illustrated using PatchDock to verify the stability of the docked complex.

To further evaluate the binding affinity between the developed vaccine construct and the relative antigenic receptors (TLR3, MHC-I, and MHC-II), molecular docking was performed. The server yielded 44 candidate models with different binding energies. Twenty-nine model complexes of TLR3 and COVID-19 vaccine were determined, from which just one complex with the lower binding energy score of −1156.2 was selected to show (Table 6 and Figure 6). A total of 29 model complexes of MHC-I and the COVID-19 vaccine were discovered, and the lowest binding energy score was −1346.8 (Table 6 and Figure 6). A total of 29 complex models of MHC-II and the COVID-19 vaccine were predicted, among which, one model complex with the lowest binding energy score of −1309.1 was chosen to show (Table 6 and Figure 6). Further, the vaccine construct was evaluated using the PatchDock server, which identified different models and produced a score table. The top 10 complexes identified were refined by the FireDock algorithm. Among those top 10 models, the model with the lowest binding energy was further selected to show in this paper. The refinement outcomes of TLR3 and the vaccine complex was solution number 1 with global energy of −38.40, attractive van der Waals energy (VdW) of −26.02, repulsive (VdW) of 8.62, and atomic contact energy of −11.06 (Table 6 and Figure 6). The complex of MHC-I and the vaccine was ranked number nine, with global energy of −22.97, attractive VdW of −26.84, repulsive VdW of 12.82, and atomic contact energy of −1.79 (Table 6 and Figure 6). The complex of MHC-II and the vaccine was ranked number three, with global energy of −27.52, attractive VdW of −26.86, repulsive VdW of 10.93, and atomic contact energy of 0.77 (Table 6 and Figure 6).

**Table 6:** Molecular docking of final vaccine constructs with TLR3, Mda5, and MHC-II.

**Receptor**	**ClusPro**		**FireDock**			
	**Center**	**Lowest energy**	**Glob^a^**	**aVdW^b^**	**rVdW^c^**	**ACE^d^**
TLR3	−1156.2	−1416.4	−38.40	−26.02	8.62	−11.06
MHC-I	−1346.8	−1379.8	−22.97	−26.84	12.82	−1.79
MHC-II	−1309.1	−1389.3	−27.52	−26.86	10.93	0.77

a *Glob, Global Energy*.

b *aVdW, attractive van der Waals energy*.

c *rVdW, repulsive van der Waals energy*.

d *ACE, atomic contact energy*.

### Molecular Dynamic Simulation

**Figure 7:**
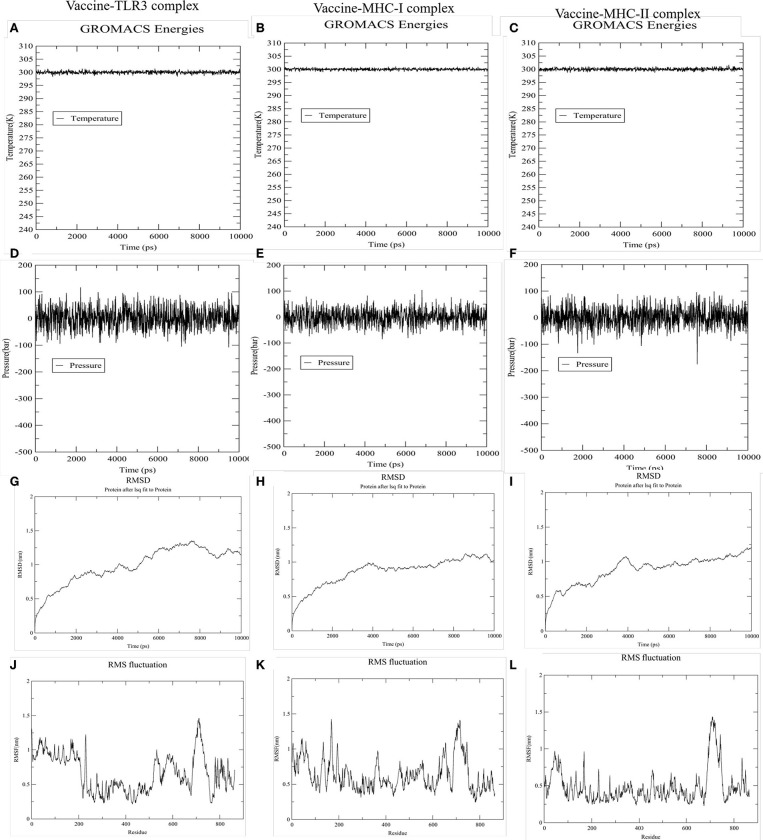
The results of molecular dynamics simulation of vaccine and immune receptors. **(A–C)** show the equilibration phase ensembles-temperature (constant at 300 k for 100 ps) of the complex of vaccine-TLR3, MHC-I, and MHC-II, respectively. **(D–F)** represent the pressure (displaying fluctuations at 1 bar value for 100 ps) of the complex of vaccine-TLR3, MHC-I, and MHC-II, respectively. **(G–I)** suggest the RMSD (root mean square deviation) plots reflect the stability between the vaccine and TLR-3, MHC-I, and MHC-II receptor, separately. Whereas, **(J–L)** RMSF (root mean square fluctuation) reflect the flexibility and fluctuation of the amino-acids residues in the side chain of docked complexes (the complex of vaccine-TLR3, MHC-I, and MHC-II), separately.

To accomplish the estimate of the stability of the vaccine-receptor complex, we performed the simulation of the docked complexes (vaccine and TLR-3, MHC-I, and MHC-II) with the help of GROMACS. Then, various analysis like energy minimization, pressure assessment, temperature, and potential energy calculations were performed. The temperature and pressure of the simulation system during the production run was around 300 K and 1 atmosphere, respectively, indicating a stable system and successful md run. The temperature and pressure of the three simulation systems (vaccine and TLR-3, MHC-I, and MHC-II complexes) during the production run were around 300 K and 1 atmosphere, respectively, indicating the stable systems and successful MD run (Figures 7A–F). The complex root mean square deviation (RMSD) plot represents the structural fluctuation of the overall structure of the complex of vaccine and immune receptor. The RMSD of vaccine-TLR3 complex has large fluctuation during 0–6 ns simulation. After 6 ns, the RMSD value was kept around 1.25 nm, indicating that the conformation of this complex was stable (Figure 7G). Otherwise, the RMSD of vaccine-MHC-I and -MHC-II complexes has large fluctuation during 0–4 ns simulation. After 4 ns, the RMSD value were kept around 1 nm, indicating that the conformation of the two complexes were stable (Figures 7H,I). Next, the root medium square fluctuation (RMSF) indicates the flexibility of the residue in the docking complex. From the results of vaccine-TLR3, MHC-I, and MHC-II complexes, residue 200–600 has low RMSF value, indicating these residues has low structural flexibility. By contrast, residue 0–200 and 600–800 has relatively higher RMSF value, indicating the larger flexibility during those regions (Figures 7J–L).

### *In silico* Cloning and Prediction of RNA Secondary Structure

**Figure 8:**
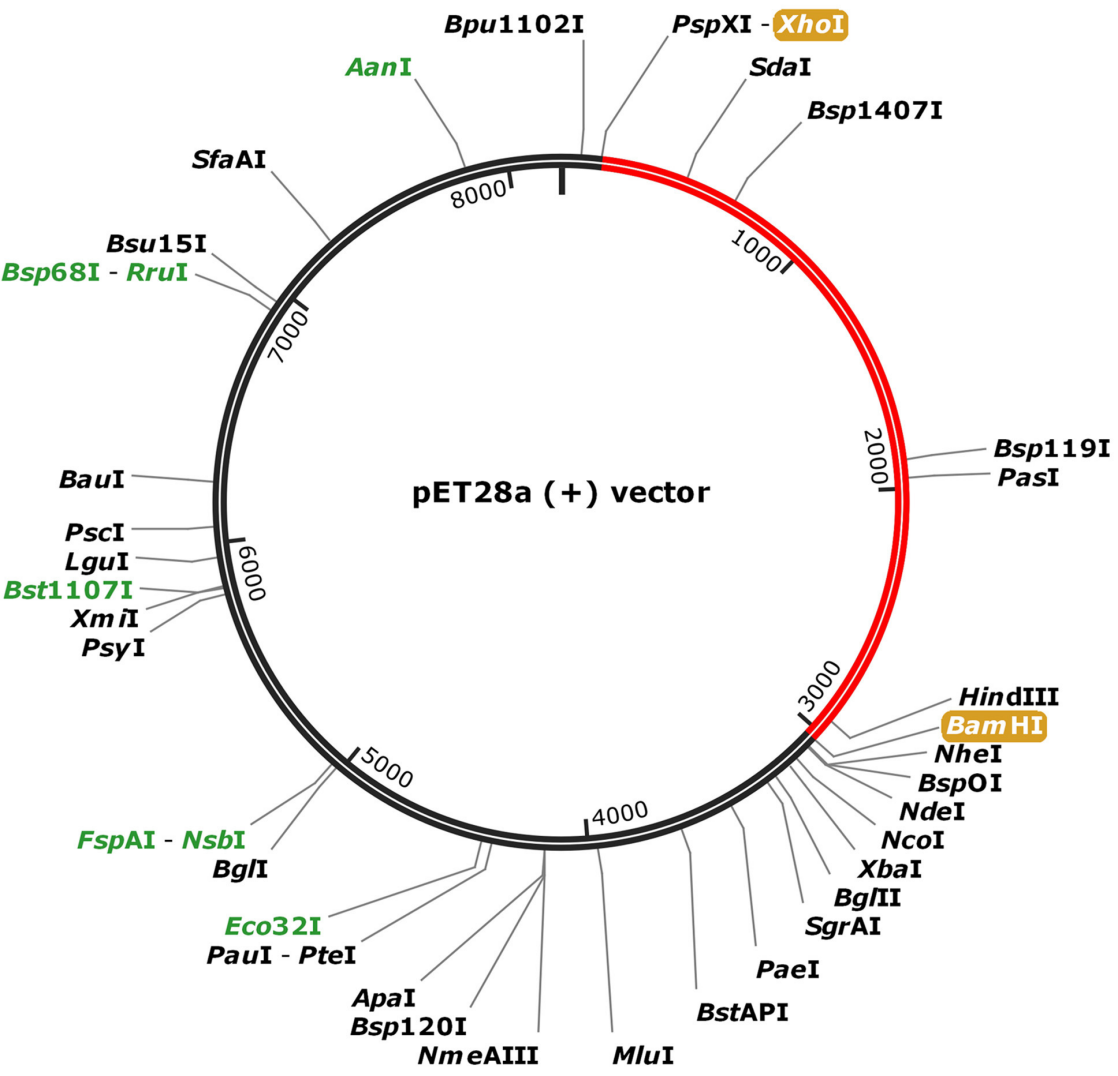
*In silico* cloning of the SARS-CoV-2 vaccine in the vector, pET28a (+). Red areas represent the COVID-19 vaccine, while the black areas represent the expression vector, pET28a (+).

To fuse the final vaccine to an expression vector, codon conversion of the vaccine protein was performed by the Java Codon Adaptation tool. Restriction site XhoI and Bam HI were added to *N* and *C* terminals of the codon sequence, then was inserted into the pET28a (+) vector between the XhoI and BamHI (Figure 8). The RNA secondary structure using the Mfold program was generated foldings contain 4,381 base pairs out of 2.3% in the energy dot plot. Mfold predicted an identical secondary structure of 4,381 bp formed by nucleotide fragments (Figure S7).

## Discussion

SARS-CoV-2 is characterized by high infectivity and high transmission speed; thus, a prophylactic vaccine is needed (11). The availability and advantages of the multi-peptide vaccine developed by immunoinformatics methods have been confirmed by previous studies (73, 74). Ojha et al. used the immunoinformatics methods to develop a multiepitope subunit vaccine to Epstein-Barr virus-associated malignancy (73). In recent studies, genomics and proteomics information of SARS-CoV-2 have been retrieved, stored, and utilized (75, 76). In the present research, we tried to develop a multi-epitope subunit prophylactic vaccine of SARS-CoV-2, with the help of immunoinformatics tools.

A line of research have tried to develop the vaccine of SARS-CoV-2 by immunoinformatics tools. Baruah and Bose (15) used immunoinformatics tools to discover cytotoxic T lymphocyte (CTL) and B cell epitopes for the spike protein of SARS-CoV-2. Then, Abraham et al. developed a multi-epitope vaccine that was designed using immunoinformatics tools that potentially trigger both CD4+ and CD8+ T-cell immune responses (16). Most of those research just focus on the spike protein-based vaccine. A vaccine based on the spike protein could induce antibodies to block SARS-COV-2 binding and fusion or neutralize virus infection (18), as well as induce harmful immune responses that cause liver damage (19). Other proteins should be ideal candidates for designing vaccines.

In the present report, we selected nine proteins with positive antigenicity for further epitope prediction. All proteins from SARS-CoV-2 with <100 amino acid sequences were excluded, and the antigenic nature of the remaining proteins was evaluated. This method can facilitate the discovery of potential antigens of SARS-CoV-2 when the precise immunity mechanisms are unknown. To design an effective vaccine, we selected the SARS-CoV-2 protein through the above-mentioned methods for epitope prediction. In recently, Asaf et al. reported that identify multiple epitopes for CD4 ^+^ 12 and CD8 ^+^ T cells based on muti-protein (77). Their protein list was the same as this in our research. In Asaf's report, they just predicted the T cell epitopes, non-B cell, B cell peptide was not predicted (77).

The B cell epitopes are antigenic determinants from the antigen that are recognized by the B cell surface membrane receptor and evoke the production of specific antibodies. The persistent challenge in immunological prediction tools is the prediction of epitopes to a higher level of accuracy (78). To determine accurate linear B cell epitopes from the antigenic proteins, we used two bioinformatics tools based on different algorithms of prediction. We identified nine overlapping linear B cell epitope candidates from two different bioinformatics tools. This method was superior to the prediction of epitopes from a single tool (78). Moreover, we also have predicted the non-continue B-cell epitopes.

The B cell immune response is preferred in the design of a vaccine. However, T cells may also elicit a strong immunoreaction. The vaccine that activates both CTLs and HTLs should be more effective than a vaccine that only targets CTL responses (79). To generate a more effective vaccine, we predicted both CTL epitopes and HTL epitopes. The T cell epitopes were decomposed fragments from the antigen presented by the MHC molecules of T cells and stimulated the production of effector T cells, immunological memory T cells, and IFN-γ. The cell-mediated immune response induced by CTLs plays a vital role in the defense against viral infections through the recognition of intracellular viral pathogens by MHC class I molecules.

In the present report, MHC-I binding epitopes were predicted by choosing A2, A3, and B7 alleles, which cover ~95% of world's population. We selected 18 CTL epitopes. The HTLs play a vital role in the antiviral immune response by producing IFN-γ. Moreover, HTLs are able to induce and maintain CTL responses. Furthermore, 14 HTLs epitopes were chosen based on both the binding capability and IFN-γ induction. Bhattacharya et al. also used the spike protein sequence predicted for MHC-I and MHC-II epitopes of SARS-CoV-2, but not predicted capability of producing IFN-γ (80). The T cell epitopes enhanced IFN-γ inducing capability, which evokes both the native and specific immune responses by activating macrophages and natural killer cells, and augmenting the response of the MHC to the antigen (81, 82).

In this study, the immunogenic epitopes from B cells, CTLs, and HTLs were chosen to develop a more valid, reliable, and effective vaccine against SARS-CoV-2. A multiepitope approach was used by splicing together epitopes with the aid of their respective linkers. To improve the immunogenicity of this multiepitope vaccine, an adjuvant β-defensin and pan-HLA DR binding epitopes (13aa) were fused to the N-terminal with the aid of an EAAAK linker, then A TAT sequence (11aa) was appended to C-terminal with the added of KK. The final vaccine constituted 864 amino acids. The allergenicity, antigenicity, and stability of the designed vaccine constructs were then evaluated. The tertiary structure of the generated vaccine was predicted by using the Phyre 2 server and then refined by the GalaxyRefine server. The binding affinity of complexes of the developed vaccine and receptors, in which TLR-3, MHC-I, and MHC-II (present on the surface of the immune cell) were confirmed by the ClusPro server was based on molecular docking.

Furthermore, to ensure the translation efficiency of the designed vaccine in a specific expression system, the mRNA of the vaccine was enhanced with the aid of the Java Codon Adaptation Tool. The restriction enzyme cutting sites of Xho? and BamH? were then appended to the *N* and *C* terminals, respectively. The vaccine sequence was subsequently cloned in pET28a (+), the expression vector. Further experimental validation of the safety and efficacy of the designed vaccine for SARS-CoV-2 is warranted.

## Data Availability Statement

All datasets presented in this study are included in the article.

## Author Contributions

RD and ZC performed the experiments. RD and YZ wrote the paper. YZ and FY edited the final version. All authors participated in the experimental design, data analysis, and agreed with the final version of the paper.

## Conflict of Interest

The authors declare that the research was conducted in the absence of any commercial or financial relationships that could be construed as a potential conflict of interest.
