# Supplementary material for: Contriving Multi-Epitope Subunit of Vaccine for COVID-19: Immunoinformatics Approaches
Source: Front Immunol. 2020 Jul 28;11:1784. doi: 10.3389/fimmu.2020.01784 (PMC7399176; doi:10.3389/fimmu.2020.01784)
Supplement: Supplementary file 1 [file Data_Sheet_1.pdf]

Supplementary Material

Contriving Multi-epitope Subunit of Vaccine for COVID-19: Immunoinformatics Approaches

***Rong Dong*[1,2,3], *Fuxun Yu*[3], *Yan Zha*[1,2,3]**

[1] Guizhou University School of Medicine, Guiyang, Guizhou Province, China

[2] Department of Nephrology, Guizhou Provincial People's Hospital, Guiyang, Guizhou Province, China

[3] NHC Key Laboratory of Pulmonary Immunological Diseases (Guizhou Provincial People's Hospital), Guiyang, Guizhou Province, China

Run title: Contriving COVID-19 Vaccine by Immunoinformatics.

Correspondence

Rong Dong: drvivid1990@163.com

ORCID

Rong Dong: 0000-0003-2372-9833

Homology Modeling SARS-COV-2 proteins

c1yo4A [NMR structure of the SARS coronavirus orf 7a coded X42 protein (clyo4A), residues 16–98] for orf 7a protein of SARS-CoV-2 (identity: 92%, coverage: 69%); d1xaka [x-ray structure of the accessory protein X4 (ofr7), residues 23–42] for orf 8 protein of SARS-CoV-2 at 1.80 angstroms resolution (identity: 30%, coverage: 15%); c1uw7A [[x-ray structure structure of the SARS coronavirus nsp9 protein (clyo4A) at 3.0 angstroms resolution, residues 1–113] for nsp9 protein of SARS-CoV-2 (identity: 97%, coverage: 99%); c6irwA [crystal structure of the human cap-specific adenosine2 methyltransferase bound to SAH, residues 258–285] for nsp6 protein of SARS-CoV-2 (identity: 29%, coverage: 9%); c4yptA [x-ray structure of three tandemly linked domains of nsp3 from murine2 hepatitis virus at 2.60 angstroms resolution, residues 675-1053] for nsp3 protein of SARS-CoV-2 (identity: 27%, coverage: 19%); c2rhbD [Crystal structure of nsp15-h234a mutant-hexamer in 2 asymmetric unit, residues 1-344] for endoRNAse of SARS-CoV-2 (identity: 88%, coverage: 99%); d2o5ha1[NMB0513-like, residues 66–115] for ORF3a protein of SARS-CoV-2 (identity: 24%, coverage: 17%); c3sc0A [crystal structure of mmachc (1–238), a human b12 processing enzyme,2 complexed with methylcobalamin, residues 5–24] for membrane glycoprotein of SARS-CoV-2 (identity: 25%, coverage: 9%); d1sska [Coronavirus RNA-binding domain, residues 23–180] for nucleocapsid phosphoprotein of SARS-CoV-2 (identity: 82%, coverage: 32%); (see for the homology models and Figure S1 for the template-target pairwise sequence alignments)

STABE1. The details of structure homology of SARS-CoV-2 proteins to human proteins

Molecule	Based PBD Model	Organism	identify	coverage
ORF7a protein	Clyo4a	SARS coronavirus	92%	69%
ORF8 protein	d1xaka	SARS coronavirus	30%	15%
nsp9	c1uw7A	SARS coronavirus	97%	99%
nsp6	c6irwA	Human	29%	9%
nsp3	c4yptA	murine2 hepatitis virus	27%	19%
endoRNAse	c2rhbD	Virtus	88%	99%
ORF3a protein	d2o5ha1	-	24%	17%
membrane glycoprotein	c3sc0A	Human	25%	9%
nucleocapsid phosphoprotein	d1sska	coronavirus	82%	37%

The ramachandran plots details of the templates used to model the structure of SARS-COV-2 proteins.

Ramachandran plots of the modeled ORF7a proteins of SARS-COV-2 showed 97.53% in the favored regions, ramachandran plots of the modeled ORF8 proteins of SARS-COV-2 showed 94.44% in the favored regions, ramachandran plots of the modeled nsp9 proteins of SARS-COV-2 showed 95.50% in the favored regions, ramachandran plots of the modeled nsp6 proteins of SARS-COV-2 showed 96.15% in the favored regions, ramachandran plots of the modeled nsp3 proteins of SARS-COV-2 showed 96.2% in the favored regions, ramachandran plots of the modeled endoRNAse proteins of SARS-COV-2 showed 99.12% in the favored regions, ramachandran plots of the modeled ORF3a protein proteins of SARS-COV-2 showed 100% in the favored regions, ramachandran plots of the modeled membrane glycoprotein of SARS-COV-2 showed 100% in the favored regions, ramachandran plots of the modeled nucleocapsid phosphoprotein of SARS-COV-2 showed 98.20% in the favored regions.

STABLE2. Ramachandran plots details of SARS-COV-2 proteins of SARS-CoV-2 proteins structure

Molecule	Template	identify	coverage	favored region	Rotamer Outliers
ORF7a protein	1yo4.1.A	92%	69%	97.53%	0
ORF8 protein	d1xaka	30%	15%	94.44%	0
nsp9	c1uw7A	97%	99%	95.50%	0
nsp6	c6irwA	29%	9%	96.15%	4.35%
nsp3	c4yptA	27%	19%	96.2%	0.3%
endoRNAse	c2rhbD	88%	99%	99.12%	0%
ORF3a protein	d2o5ha1	24%	17%	100%	2.3%
membrane glycoprotein	c3sc0A	25%	9%	100.00%	5.26%
nucleocapsid phosphoprotein	d1sska	82%	37%	98.20%	0.0%

SFIGRE1

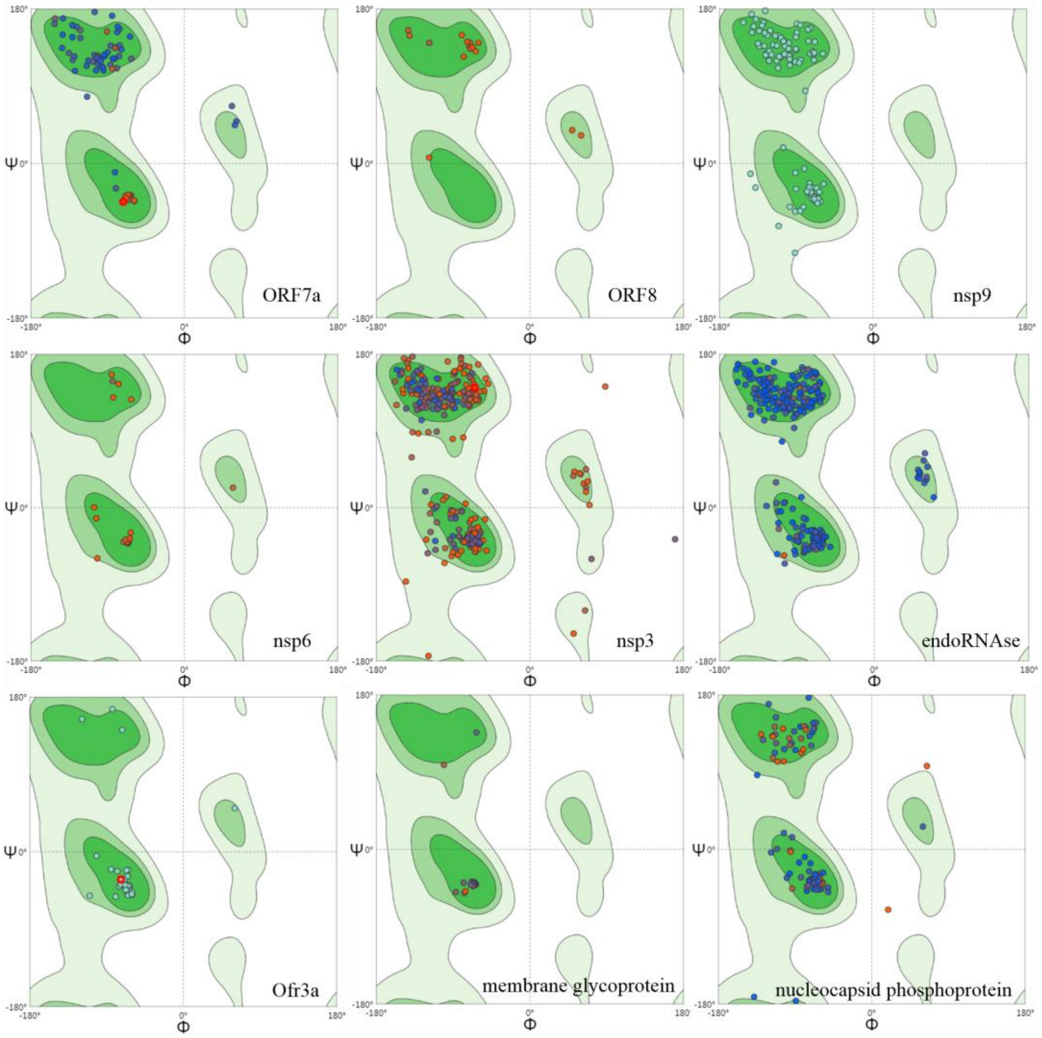

SFIGURE1: Ramachandran plots indicating the quality of the 3D structures of the SARS-COV-2 protein models.

SFIGRE2

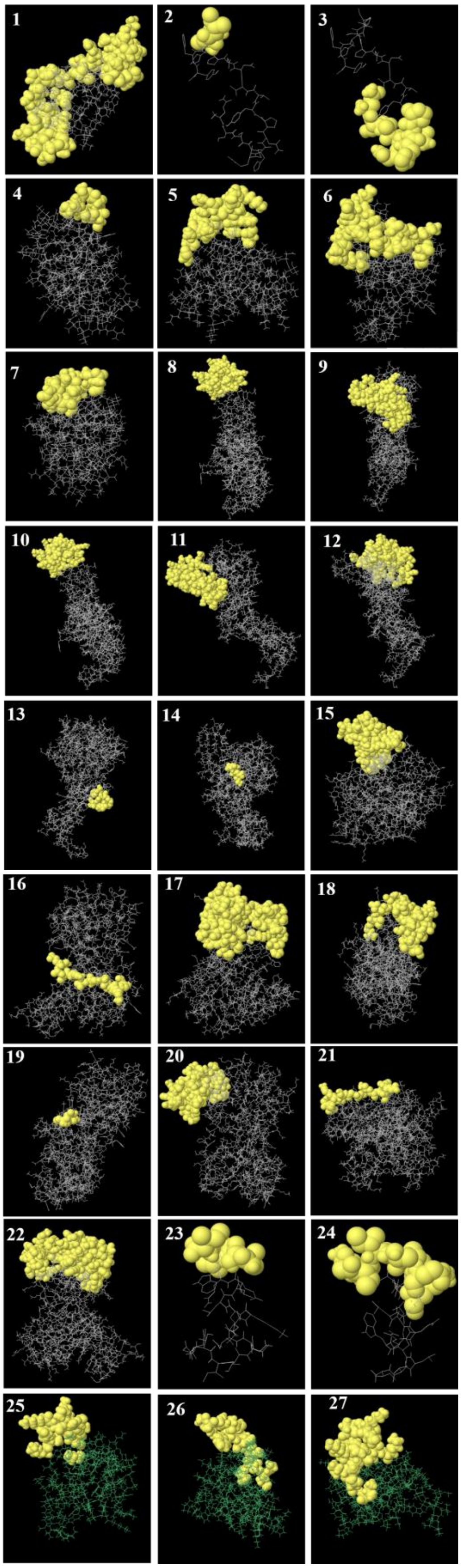

FIGURE 2. ElliPro predicted discontinuous B-cell epitopes of SARS-COV-2

represented in 3D. The numbers of the predicted epitope regions for each protein correspond to the number of prediction in TABLE5.

Sequence of Vaccine Construction

EAAAKKAKFVAAWTLKAAAEAAAKGIINTLQKYYCRVRGGRCAVLSCLPKEEQIGKCSTRGRKCCRRKKEAAAKAAYKLFIRQEEVAAYTLCFTLKRKAAYAAYSPIFLIVAAAAYALLSDLQDLAAYFLLPSLATVAAYSAFAMMFVKAAYMPASWVMRIAAYVMYMGTLSYAAYLLLDDFVEIAAYSPFGHSLTLAAYIMRLWLCWKAAYLSYFIASFRAAYLPKEITVATAAYLLLDRLNQLAAYKTFPPTEPKAAYFPRGQGVPIAAYIPIQASLPFAAYGLMWLSYFIGPGPGHFYSKWYIRVGARKSGPGPGIHFYSKWYIRVGARKGPGPGKGLNNLNRGMVLGSLGPGPGTAFGLVAEWFLAYILGPGPGAAIMQLFFSYFAVHFGPGPGGLAKRFKESPFELEDGPGPGACFVLAAVYRINWITGPGPGKLIFLWLLWPVTLACGPGPGDDQIGYYRRATRRIRGPGPGGKMKDLSPRWYFYYLGPGPGDFLEYHDVRVVLDFIGPGPGRGTTLKEPCSSGPHPLADNKCCPDGVRSVSPKLFIREEELKKSSKTPEEHFIETISLAGSYKDWSYSGQSTQLGIEFLKRGDKSVYYTSNPTTFHLDGEVITFDNLLRKKHCNLLLLFKKSGTYEGNSPFHPLADNKKKSPIQYIDIGNYTVSCKKHFYSKWYIRVGARKSAKKKGPKVKYLYFIKGLNNKKAGTTQTACTDDNALAYKKDFLELAMDEFIERYKLKKTSPISEHDYQIGGYTEKKHVQIHTIDGSSGVVNPKKYRIGNYKLNTDHSSSKKNGTITVEELKKLLEKKKSAAEASKKPRQKRTAKKEGALNTPKDHIGTRNPKKTGALLAAGAAAKK

SFIGURE 3. The sequence of 2019nCOV vaccine construct

BLAST ® » **blastp suite** » results for RID-DEKUD0VG014

Your search is limited to records that include: Humo sapiens (taxid:9606)

Job Title	Protein Sequence ...
RID	DEKUD0VG014 Search expires on 06-04 15:20 pm
Program	BLASTP
Database	nr
Query ID	lcl|Query_37817
Description	None ...
Molecule type	amino acid
Query Length	864

Descriptions

Description	Max Score	Total Score	Query Cover	E value	Per. Ident	Accession
beta-defensin 3 [Homo sapiens]	104	104	5%	3e-26	100.00%	AAV41025.1
beta-defensin-like protein [Homo sapiens]	104	104	5%	9e-26	93.88%	ACK99045.1
beta-defensin 103 precursor [Homo sapiens]	103	103	5%	2e-25	100.00%	NP_001075020.1
beta-defensin-3 [Homo sapiens]	97.8	97.8	5%	1e-23	97.78%	AAM62424.1

Graphic Summary

Alignments

Taxonomy

SFIGURE 4. The analysis of sequence homology of 2019nCOV vaccine construct

Email	drvivid1990@163.com
Description	VACCINE3_
Date	Wed May 27 04:25:09 BST 2020
Unique Job ID	a6c2868e1824a367

Detailed template information

#	Template	Alignment Coverage	3D Model	Confidence	% i.d.	Template Information
1	d1kj6a_	Alignment	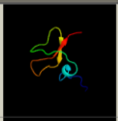	99.8	100	**Fold:**Defensin-like<br>**Superfamily:**Defensin-like<br>**Family:**Defensin
2	c2kafA_	Alignment	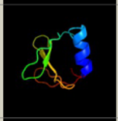	99.7	73	**PDB header:**viral protein, rna binding protein<br>**Chain:** A: **PDB Molecule:**non-structural protein 3;<br>**PDBTitle:** solution structure of the sars-unique domain-c from the nonstructural2 protein 3 (nsp3) of the severe acute respiratory syndrome coronavirus
3	c2lxoA_	Alignment	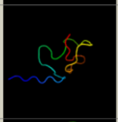	99.6	58	**PDB header:**antimicrobial protein, de novo protein<br>**Chain:** A: **PDB Molecule:**chimeric peptide;<br>**PDBTitle:** identification of the structural traits mediating the antimicrobial2 activity of a chimeric peptide of hbd2 and hbd3
4	c3hd4A_	Alignment	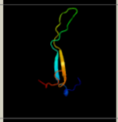	98.7	50	**PDB header:**viral protein<br>**Chain:** A: **PDB Molecule:**nucleoprotein;<br>**PDBTitle:** mhv nucleocapsid protein ntd
5	d1sska_	Alignment	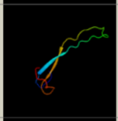	98.7	68	**Fold:**Coronavirus RNA-binding domain<br>**Superfamily:**Coronavirus RNA-binding domain<br>**Family:**Coronavirus RNA-binding domain
6	d2bxxa1	Alignment	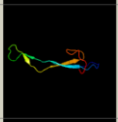	98.4	39	**Fold:**Coronavirus RNA-binding domain<br>**Superfamily:**Coronavirus RNA-binding domain<br>**Family:**Coronavirus RNA-binding domain
7	c4ud1B_	Alignment	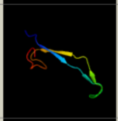	98.4	48	**PDB header:**viral protein<br>**Chain:** B: **PDB Molecule:**n protein;<br>**PDBTitle:** structure of the n terminal domain of the mers cov nucleocapsid
8	d2geca1	Alignment	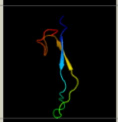	98.4	45	**Fold:**Coronavirus RNA-binding domain<br>**Superfamily:**Coronavirus RNA-binding domain<br>**Family:**Coronavirus RNA-binding domain
9	c5n4kA_	Alignment	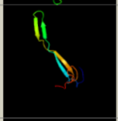	98.4	43	**PDB header:**rna binding protein<br>**Chain:** A: **PDB Molecule:**nucleoprotein;<br>**PDBTitle:** n-terminal domain of a human coronavirus nl63 nucleocapsid protein
10	c1yo4A_	Alignment	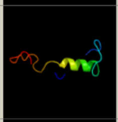	98.2	85	**PDB header:**viral protein<br>**Chain:** A: **PDB Molecule:**hypothetical protein x4;<br>**PDBTitle:** solution structure of the sars coronavirus orf 7a coded x42 protein
11	d1xaka_	Alignment	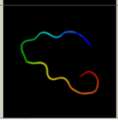	97.6	100	**Fold:**Immunoglobulin-like beta-sandwich<br>**Superfamily:**Accessory protein X4 (ORF8, ORF7a)<br>**Family:**Accessory protein X4 (ORF8, ORF7a)

12	c1uw7A_	Alignment	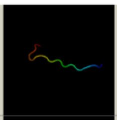	95.9	100	**PDB header:**viral protein<br>**Chain:** A: **PDB Molecule:**nsp9;<br>**PDBTitle:** nsp9 protein from sars-coronavirus.
13	d1qz8a_	Alignment	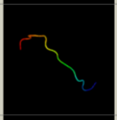	95.9	100	**Fold:**Replicase NSP9<br>**Superfamily:**Replicase NSP9<br>**Family:**Replicase NSP9
14	c2j98A_	Alignment	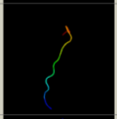	94.9	69	**PDB header:**rna binding protein<br>**Chain:** A: **PDB Molecule:**replicase polyprotein 1ab;<br>**PDBTitle:** human coronavirus 229e non structural protein 9 cys69ala mutant (nsp9)
15	c5hizA_	Alignment	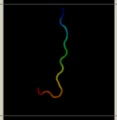	94.5	50	**PDB header:**rna binding protein<br>**Chain:** A: **PDB Molecule:**non-structural protein 9;<br>**PDBTitle:** the structure of pedv nsp9
16	c2rhbD_	Alignment	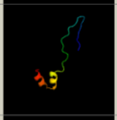	93.2	38	**PDB header:**viral protein<br>**Chain:** D: **PDB Molecule:**uridylate-specific endoribonuclease;<br>**PDBTitle:** crystal structure of nsp15-h234a mutant- hexamer in2 asymmetric unit
17	d2ozka2	Alignment	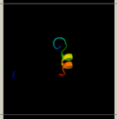	92.4	79	**Fold:**EndoU-like<br>**Superfamily:**EndoU-like<br>**Family:**Nsp15 C-terminal domain-like
18	d2h85a2	Alignment	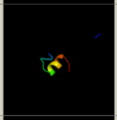	92.4	79	**Fold:**EndoU-like<br>**Superfamily:**EndoU-like<br>**Family:**Nsp15 C-terminal domain-like
19	c5ym6B_	Alignment	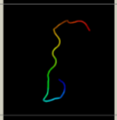	91.6	35	**PDB header:**rna binding protein<br>**Chain:** B: **PDB Molecule:**nsp9;<br>**PDBTitle:** crystal structure of porcine delta coronavirus nsp9
20	d2cjra1	Alignment	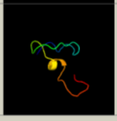	81.0	57	**Fold:**Nucleocapsid protein dimerization domain<br>**Superfamily:**Nucleocapsid protein dimerization domain<br>**Family:**Coronavirus nucleocapsid protein
21	c5yvdA_	Alignment	not modelled	79.0	37	**PDB header:**hydrolase<br>**Chain:** A: **PDB Molecule:**nsp15;<br>**PDBTitle:** structural and biochemical characterization of endoribonuclease nsp152 encoded by middle east respiratory syndrome coronavirus
22	d1fd3a_	Alignment	not modelled	77.1	36	**Fold:**Defensin-like<br>**Superfamily:**Defensin-like<br>**Family:**Defensin
23	c4rs4A_	Alignment	not modelled	74.5	26	**PDB header:**hydrolase<br>**Chain:** A: **PDB Molecule:**uridylate-specific endoribonuclease;<br>**PDBTitle:** crystal structure and mutational analysis of the endoribonuclease from2 human coronavirus 229e
24	d1bnba_	Alignment	not modelled	67.3	44	**Fold:**Defensin-like<br>**Superfamily:**Defensin-like<br>**Family:**Defensin
25	c2drtB_	Alignment	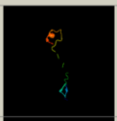	64.7	100	**PDB header:**structural protein<br>**Chain:** B: **PDB Molecule:**collagen like peptide;<br>**PDBTitle:** structure analysis of (pog)4-log-(pog)5
26	c2drtA_	Alignment	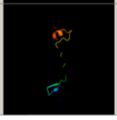	64.7	100	**PDB header:**structural protein<br>**Chain:** A: **PDB Molecule:**collagen like peptide;<br>**PDBTitle:** structure analysis of (pog)4-log-(pog)5

27	c2mqsD_	Alignment	not modelled	64.4	48	**PDB header:**hydrolase<br>**Chain:** D: **PDB Molecule:**thp_t_chain;<br>**PDBTitle:** transient collagen triple helix binding to a key metalloproteinase in2 invasion and development: spin labels to structure
28	c2l3aA_	Alignment	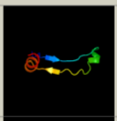	63.3	27	**PDB header:**structural genomics, unknown function<br>**Chain:** A: **PDB Molecule:**uncharacterized protein;<br>**PDBTitle:** solution nmr structure of homodimer protein sp_0782 (7-79) from2 streptococcus pneumoniae northeast structural genomics consortium3 target spr104 .
29	c3obhA_	Alignment	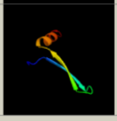	62.9	28	**PDB header:**structural genomics, unknown function<br>**Chain:** A: **PDB Molecule:**uncharacterized protein;<br>**PDBTitle:** x-ray crystal structure of protein sp_0782 (7-79) from streptococcus2 pneumoniae. northeast structural genomics consortium target spr104
30	c2mqsC_	Alignment	not modelled	60.7	48	**PDB header:**hydrolase<br>**Chain:** C: **PDB Molecule:**thp_l_and_m_chain;<br>**PDBTitle:** transient collagen triple helix binding to a key metalloproteinase in2 invasion and development: spin labels to structure
31	c2mqsB_	Alignment	not modelled	60.7	48	**PDB header:**hydrolase<br>**Chain:** B: **PDB Molecule:**thp_l_and_m_chain;<br>**PDBTitle:** transient collagen triple helix binding to a key metalloproteinase in2 invasion and development: spin labels to structure
32	c2ltdA_	Alignment	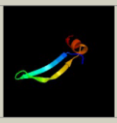	59.5	33	**PDB header:**structural genomics, unknown function<br>**Chain:** A: **PDB Molecule:**uncharacterized protein ydbc;<br>**PDBTitle:** solution nmr structure of apo ydbc from lactococcus lactis, northeast2 structural genomics consortium (nesg) target kr150
33	c6hg7A_	Alignment	not modelled	58.9	52	**PDB header:**structural protein<br>**Chain:** A: **PDB Molecule:**collagen alpha-1(ii) chain;<br>**PDBTitle:** crystal structure of a collagen ii fragment containing the binding2 site of pedf and comp, (pog)4-lkg hrg ftg lqg-pog(4)
34	c6hg7C_	Alignment	not modelled	58.9	52	**PDB header:**structural protein<br>**Chain:** C: **PDB Molecule:**collagen alpha-1(ii) chain;<br>**PDBTitle:** crystal structure of a collagen ii fragment containing the binding2 site of pedf and comp, (pog)4-lkg hrg ftg lqg-pog(4)
35	c6hg7B_	Alignment	not modelled	58.9	52	**PDB header:**structural protein<br>**Chain:** B: **PDB Molecule:**collagen alpha-1(ii) chain;<br>**PDBTitle:** crystal structure of a collagen ii fragment containing the binding2 site of pedf and comp, (pog)4-lkg hrg ftg lqg-pog(4)
36	c6jklC_	Alignment	not modelled	57.8	67	**PDB header:**structural protein<br>**Chain:** C: **PDB Molecule:**a triple-helix region of human collagen type ii;<br>**PDBTitle:** structure of a triple-helix region of human collagen type ii
37	c6jklA_	Alignment	not modelled	57.8	67	**PDB header:**structural protein<br>**Chain:** A: **PDB Molecule:**a triple-helix region of human collagen type ii;<br>**PDBTitle:** structure of a triple-helix region of human collagen type ii
38	c6jklB_	Alignment	not modelled	57.8	67	**PDB header:**structural protein<br>**Chain:** B: **PDB Molecule:**a triple-helix region of human collagen type ii;<br>**PDBTitle:** structure of a triple-helix region of human collagen type ii
39	c2drxA_	Alignment	not modelled	57.6	100	**PDB header:**structural protein<br>**Chain:** A: **PDB Molecule:**collagen like peptide;<br>**PDBTitle:** structure analysis of (pog)4-(log)2-(pog)4
40	c2drxB_	Alignment	not modelled	57.4	100	**PDB header:**structural protein<br>**Chain:** B: **PDB Molecule:**collagen like peptide;<br>**PDBTitle:** structure analysis of (pog)4-(log)2-(pog)4
41	c3pm7A_	Alignment	not modelled	57.3	33	**PDB header:**structural genomics, unknown function<br>**Chain:** A: **PDB Molecule:**uncharacterized protein;<br>**PDBTitle:** crystal structure of ef_3132 protein from enterococcus faecalis at the2 resolution 2a, northeast structural genomics consortium target efr184
42	d2ca1a1	Alignment	not modelled	57.2	29	**Fold:**Nucleocapsid protein dimerization domain<br>**Superfamily:**Nucleocapsid protein dimerization domain<br>**Family:**Coronavirus nucleocapsid protein
43	c2drxC_	Alignment	not modelled	55.6	100	**PDB header:**structural protein<br>**Chain:** C: **PDB Molecule:**collagen like peptide;<br>**PDBTitle:** structure analysis of (pog)4-(log)2-(pog)4
44	d1ut3a_	Alignment	not modelled	55.3	50	**Fold:**Defensin-like<br>**Superfamily:**Defensin-like<br>**Family:**Defensin
45	c1ut3A_	Alignment	not modelled	55.3	50	**PDB header:**antibiotic<br>**Chain:** A: **PDB Molecule:**spheniscin-2;<br>**PDBTitle:** solution structure of spheniscin-2, a beta-defensin from2 penguin stomach preserving food
46	d1e4qa_	Alignment	not modelled	53.2	36	**Fold:**Defensin-like<br>**Superfamily:**Defensin-like<br>**Family:**Defensin
47	c3ponB_	Alignment	not modelled	52.6	81	**PDB header:**unknown function<br>**Chain:** B: **PDB Molecule:**mbl collagen-like peptide;<br>**PDBTitle:** crystal structure of mbl collagen-like peptide
48	c3pobC_	Alignment	not modelled	52.6	81	**PDB header:**hydrolase<br>**Chain:** C: **PDB Molecule:**mbl collagen-like peptide;<br>**PDBTitle:** crystal structure of masp-1 cub2 domain in complex with the collagen-2 like domain of mbl
						**PDB header:**unknown function

SFIGURE 5. The templates were employed modeling of vaccine structure

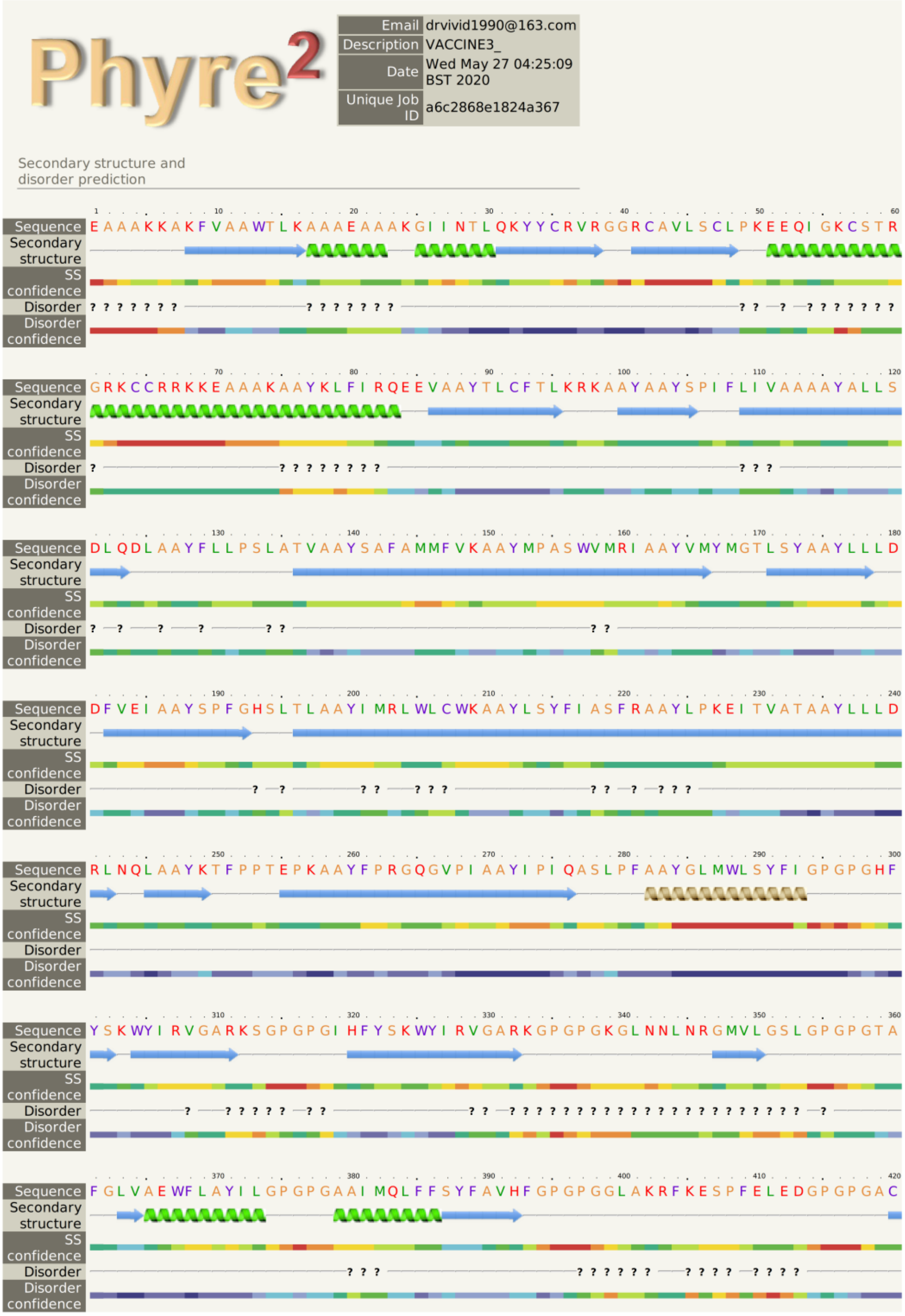
Phyre2
Email drvivid1990@163.com
Description VACCINE3_
Date Wed May 27 04:25:09 BST 2020
Unique Job ID a6c2868e1824a367
Secondary structure and disorder prediction
Sequence
Secondary structure
SS confidence
Disorder
Disorder confidence
1 EAAAKKAKFVAAWTLKAAAEAAAKGIINTLQKYYCRVRGGRCAVLSCLPKEEQIGKCSTR 60
GRKCCRRKKEAAAKAAYKLFIRQEEVAAYTLCFTLKRKAAYAAYSPIFLIVAAAAYALLS 120
DLQDLAAYFLLPSLATVAAYSAFAMMFVKAAYMPASWVMRIAAYVMYMGTLSYAAYLLLD 180
DFVEIAAYSPFGHSLTLAAYIMRLWLCWKAAYLSYFIASFRAAYLPKEITVATAAYLLLD 240
RLNQLAAYKTFPPTEPKAAYFPRGQGVPIAAYIPIQASLPFAAYGLMWLSYFIGPGPGHF 300
YSKWYIRVGARKSGPGPGIHFYSKWYIRVGARKGPGPGKGLNNLNRGMVLGSLGPGPGTA 360
FGLVAEWFLAYILGPGPGAAIMQLFFSYFAVHFGPGPGGLAKRFKESPFELEDGPGPGAC 420

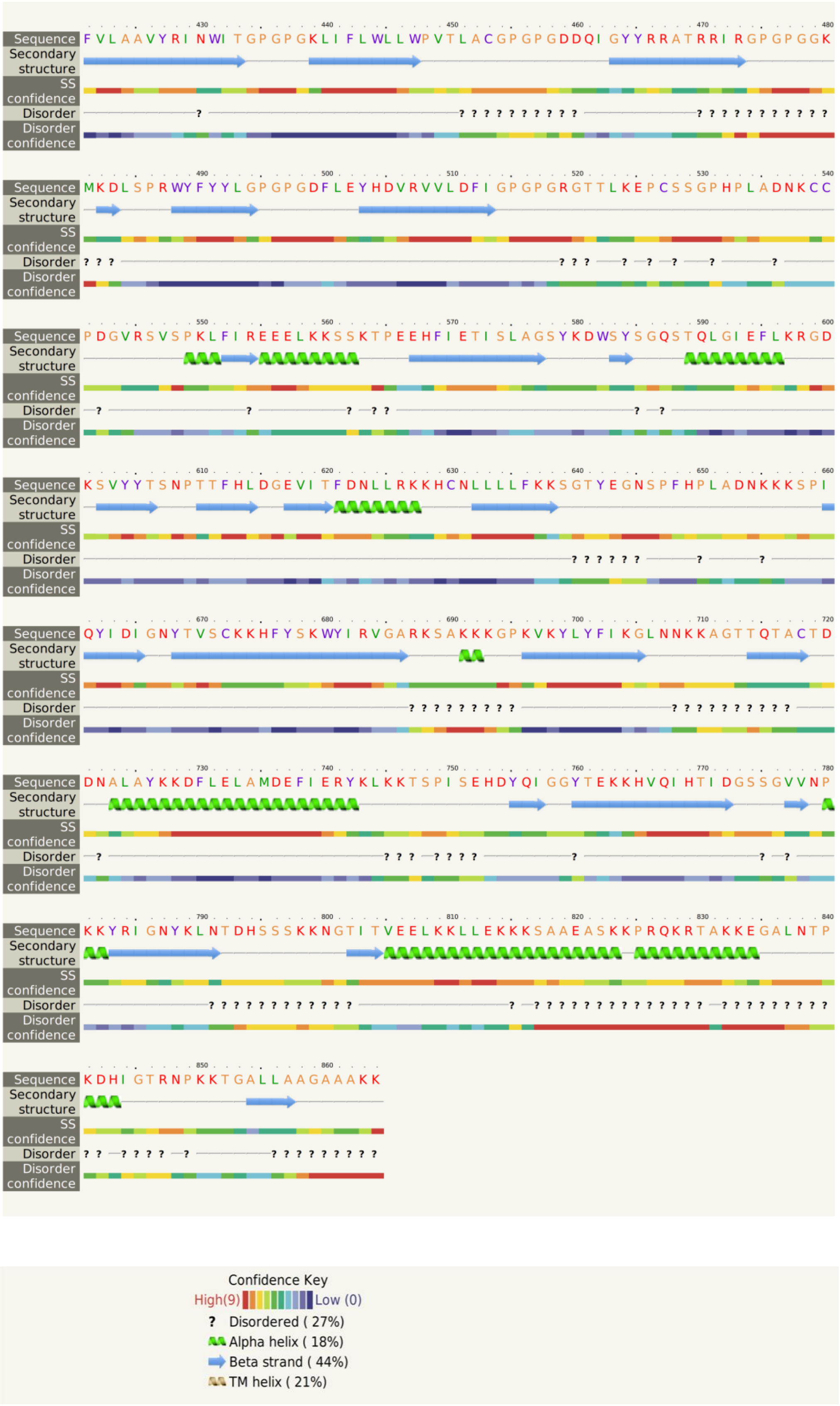

SFIGURE 6. The disorder regions and profile of constructed vaccine

STABLE3. The details of five candidate models generated from the GalaxyRefine server

Model	GDT	RMSD	MolProbity	Clashscore	Poor rotamer	Ramafavore
Initial	1.0000	0.000	4.325	172.6	9.3	63.5
MODEL 1	0.9922	0.260	2.049	8.9	0.3	89.1
MODEL 2	0.9933	0.260	2.052	9.0	0.3	89.2
MODEL 3	0.9957	0.234	2.107	10.1	0.3	88.6
MODEL 4	0.9916	0.246	2.081	9.7	0.4	89.1
MODEL 5	0.9896	0.268	2.033	8.6	0.3	89.2

GDT: global distance test; RMSD: The Root-mean-square deviation.

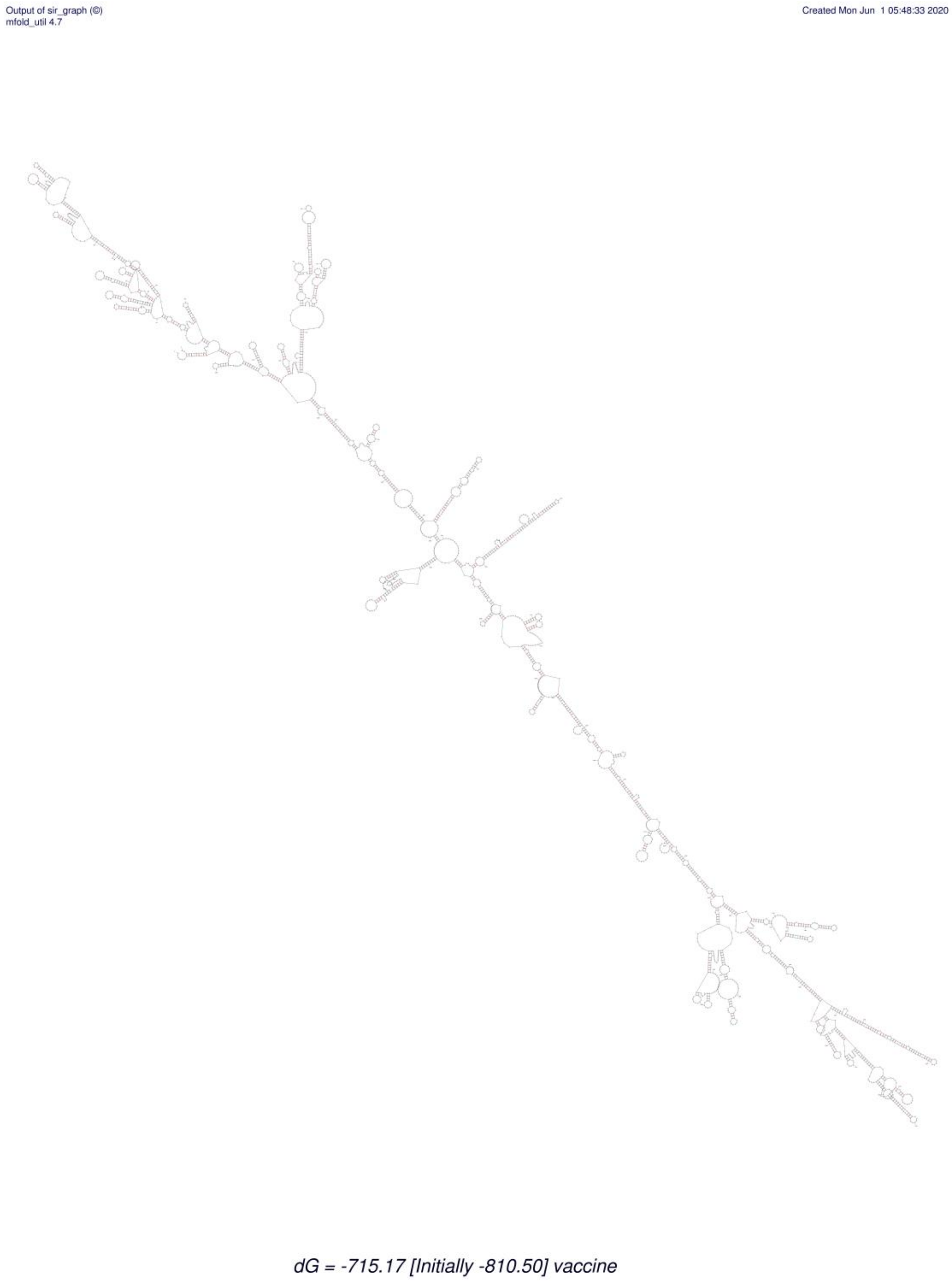

SFIGURE 7. The secondary structure of constructed vaccine mRNA
